# Enhanced Inhibition of *Fusarium graminearum* Growth and Deoxynivalenol Production by Quinolizidine Alkaloids Combined with Berberine: In Laboratory Culture Media and Pig Feed

**DOI:** 10.3390/toxins18080348

**Published:** 2026-08-15

**Authors:** Mengyang Li, Penghua Jian, Jiayi Liu, Yiwei Jia, Hui Deng, Lingxian Yi, Nan Zhuang, Zelin Hong, Daojin Yu, Bo Yang

**Affiliations:** Fujian Key Laboratory of Traditional Chinese Veterinary Medicine and Animal Health, College of Animal Sciences, Fujian Agriculture and Forestry University, Fuzhou 350002, China

**Keywords:** matrine, sophoridine, sophocarpine, berberine hydrochloride, *Fusarium graminearum*, deoxynivalenol

## Abstract

Phytochemicals with antifungal activity against *Fusarium graminearum* (*F. graminearum*) are considered potential in-feed anti-mycotoxigenic agents that may complement conventional post-harvest control strategies. Therefore, we screened fourteen phytochemicals, including seven quinolizidine alkaloids (QAs), for anti-*F. graminearum* activity, evaluated their individual and combined efficacy in inhibiting mycelial growth, spore germination, and deoxynivalenol (DON) production, and explored the underlying mechanisms via transcriptomic analysis. Matrine (MT), sophoridine (SR), and sophocarpine (SC) exhibited antifungal activity against *F. graminearum*, with minimum inhibitory concentrations (MICs) of 5, 4, and 4 mg/mL, respectively. The three QAs at their MICs completely inhibited mycelial growth and DON production in liquid cultures, but were less effective on potato dextrose agar plates and in pig feed. Particularly pronounced antifungal activity and inhibitory effects on DON production were observed when the three QAs were applied in combination with berberine hydrochloride (BBR). The observed inhibition appears to be attributed to dysregulated expression of the *TRI* cluster genes and *ERG* biosynthesis genes, coupled with significant modulation of steroid biosynthesis (ko00100) and central carbon/lipid metabolism pathways. These findings provide preliminary experimental evidence supporting the further exploration of QA–BBR combinations to control fungal proliferation and DON accumulation in pig feed.

## 1. Introduction

*Fusarium graminearum* (*F. graminearum*) is a phytopathogenic fungus that primarily infects cereal crops such as wheat and maize, causing fusarium head blight and stalk rot [1]. Notably, this pathogen produces deoxynivalenol (DON, CAS 51481-10-8), a secondary metabolite and the predominant trichothecene mycotoxin found in contaminated wheat, maize, and other cereal grains [2]. Given that these cereals serve as key ingredients in compound feed for livestock and poultry, DON contamination poses a direct threat to animal health and welfare, and undermines production profitability. Epidemiological surveillance underscores the scale of this problem. Globally, DON contamination in cereal-based feeds is widespread and persistent [3]. Surveillance data from China indicate that DON was detected in 83.3–100% of cereal-based feed raw materials, with mean concentrations ranging from 525.6 to 2186.8 μg/kg [4]. Meanwhile, liquid feeding has been increasingly adopted in intensive pig production because it improves feed efficiency and benefits animal health [5,6]. Nevertheless, poor management practices (e.g., inadequate pipeline cleaning or inappropriate water-to-feed ratios) facilitate fungal proliferation and DON accumulation [7], increasing the risk of dietary DON exposure in livestock and poultry. Once ingested, DON can induce a range of adverse effects, including reduced feed intake, impaired intestinal barrier function, immune dysfunction, growth retardation, and compromised reproductive performance [8,9]. Among livestock species, pigs are the most sensitive to DON due to its high oral bioavailability and relatively long elimination half-life, as well as the absence of intestinal microbes capable of degrading the toxin [10]. Thus, controlling DON contamination in feed and mitigating its toxicity in animals, particularly in pigs, represents a critical challenge and research priority for the livestock production sector. 

Various methods have been developed to reduce or eliminate DON in feed. These methods, which primarily focus on post-harvest detoxification, fall into three categories: physical, chemical, and biological [11]. Specific examples include sorting, washing, heat treatment, ozone application, the use of adsorbents/binders, and enzymatic biotransformation, among others. Despite this diversity, practical application of these methods is limited by several drawbacks, including low detoxification efficacy against DON [12], potential adverse effects on feed quality and nutritional value [12,13,14], and the high cost of required cofactors in some cases [11]. Given the drawbacks of these post-harvest detoxification methods, a complementary strategy is to inhibit *F. graminearum* growth and DON production at the source. Certain phytochemicals have shown promising antifungal activity against *Fusarium* species and could be incorporated into pig feed to mitigate DON contamination. However, their suitability as practical feed additives varies considerably depending on multiple factors such as chemical stability, production cost, and water solubility. For instance, eugenol, carvacrol, and thymol, which can disrupt ribosome and mitochondrial function as well as enzyme synthesis, have demonstrated efficacy against *F. graminearum* [15]. However, the high volatility and chemical instability of these phenolic compounds can compromise their antifungal activity during storage and processing of feed. The imidazole derivative of rutaecarpine A1 from *Evodia rutaecarpa* induces mitochondrial distortion and swelling, thereby inhibiting sclerotia formation and germination in *F. graminearum* [16]. Its suitability as a feed additive, however, remains to be evaluated. Canthin-6-one from *Ailanthus altissima* inhibits *Fusarium oxysporum* f. sp. *Cucumerinum* via oxidative damage to the cell membrane [17], while its potential for use as a feed additive is limited by practical considerations such as high production cost and low water solubility. Cinnamaldehyde inhibits *Fusarium sambucinum* by reducing ergosterol content and disrupting membrane function [18], but it suffers from the same drawbacks of high volatility and chemical instability. Of note, quinolizidine alkaloids (QAs) from *Lupinus* species significantly inhibit mycelial growth of *Fusarium oxysporum* [19,20], though their mechanism of action remains unclear. Building on this reported activity and considering the conserved cellular biology within the *Fusarium genus*, we hypothesized that QAs may also target *F. graminearum*. Moreover, unlike many antifungal phytochemicals mentioned above, QAs exhibit desirable water solubility and chemical stability. These physicochemical properties enable sufficient exposure of fungi to QAs, making such alkaloids attractive candidates for future antifungal application in animal feeds, particularly in liquid feeding systems.

Based on these considerations, we screened fourteen phytochemicals, including seven QAs, for their ability to inhibit *F. graminearum* growth and DON production. The most effective candidates were then evaluated, both individually and in combination, for their ability to inhibit fungal proliferation and subsequent DON accumulation in pig feed. Furthermore, preliminary investigations were conducted to explore the underlying mechanisms. Importantly, all experiments in the present study were performed in laboratory culture media and inoculated pig feed matrices, rather than under practical on-farm liquid feeding conditions. Further trials are therefore required to evaluate the practical application potential of these alkaloids in real liquid feeding systems.

## 2. Results

### 2.1. Inhibitory Activities of Fourteen Phytochemicals Against F. graminearum Strain PH-1 and Enhanced Antifungal Effects of Selected Binary Combinations

Among the fourteen tested phytochemicals, only three exhibited anti-fungal activity against *F. graminearum* strain PH-1. Specifically, matrine (MT, CAS 519-02-8) had a minimum inhibitory concentration (MIC) of 5 mg/mL, whereas sophoridine (SR, CAS 6882-68-4) and sophocarpine (SC, CAS 6483-15-4) both had MICs of 4 mg/mL. The minimal fungicidal concentrations (MFCs) for the three active QAs were identical to their MICs: 5 mg/mL for MT, and 4 mg/mL for both SR and SC. In contrast, the remaining eleven phytochemicals showed no antifungal activity, as indicated by turbid broth and normal mycelial growth at their maximum soluble concentrations. Notably, enhanced antifungal activity was observed when berberine hydrochloride (BBR, CAS 633-65-8) at 4 mg/mL was combined individually with sub-inhibitory concentrations (0.5× MIC) of MT, SR, or SC.

### 2.2. Inhibitory Effects on F. graminearum Growth and DON Production by QAs and QA-BBR Combinations

**Figure 1:**
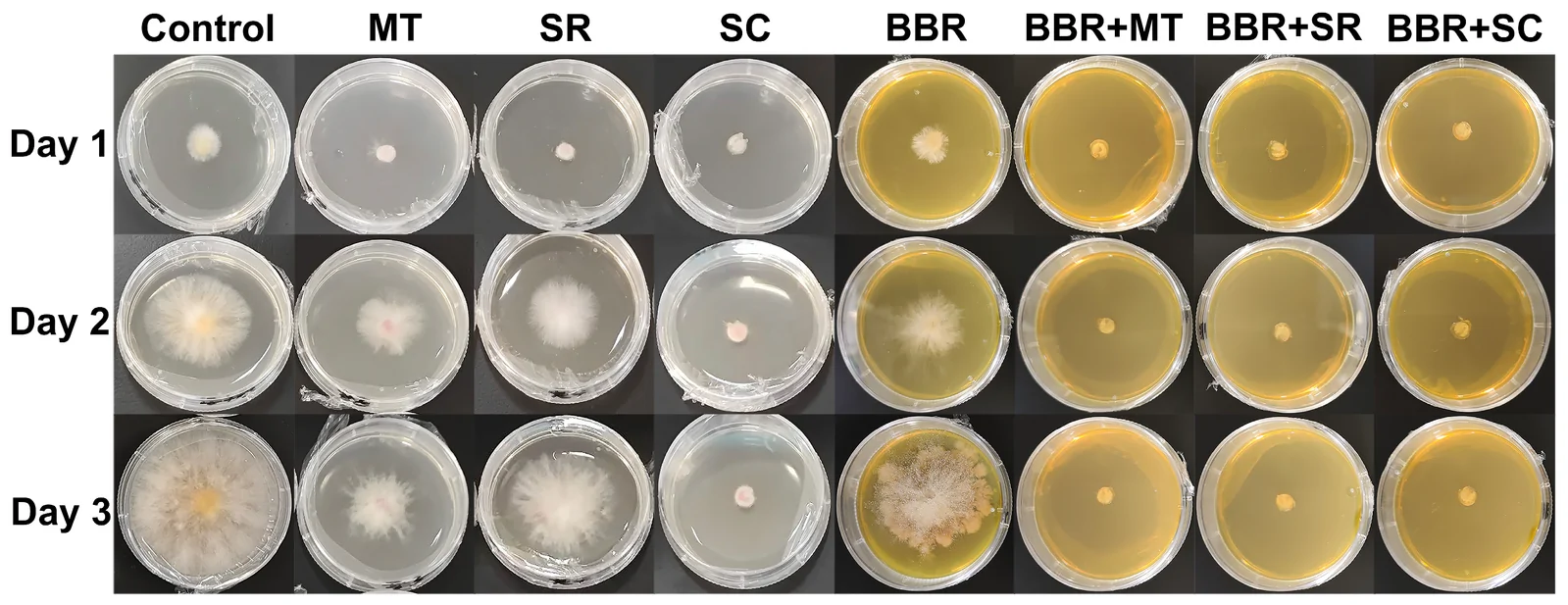
Inhibitory effects of MT, SR, SC, and BBR, either individually or in combination, on mycelial growth of *F. graminearum* strain PH-1 on PDA plates.

Figure 1 shows the inhibitory effects of MT, SR, SC, and BBR on mycelial growth of *F. graminearum* strain PH-1 on potato dextrose agar (PDA) plates. The untreated control exhibited normal mycelial expansion. MT (5 mg/mL), SR (4 mg/mL), SC (4 mg/mL), BBR (4 mg/mL), and combinations of each QA with BBR (4 mg/mL) inhibited mycelial growth based on visual assessment. Clear differences in mycelial expansion were observed among treatments. Among the three QAs, visual assessment indicated that SC exhibited stronger antifungal activity than MT and SR, while SR alone showed only weak inhibitory activity. Although BBR alone exhibited only weak inhibitory activity, its combination with either MT or SR result in more pronounced inhibition of mycelial growth compared to the respective QA alone.

**Figure 2:**
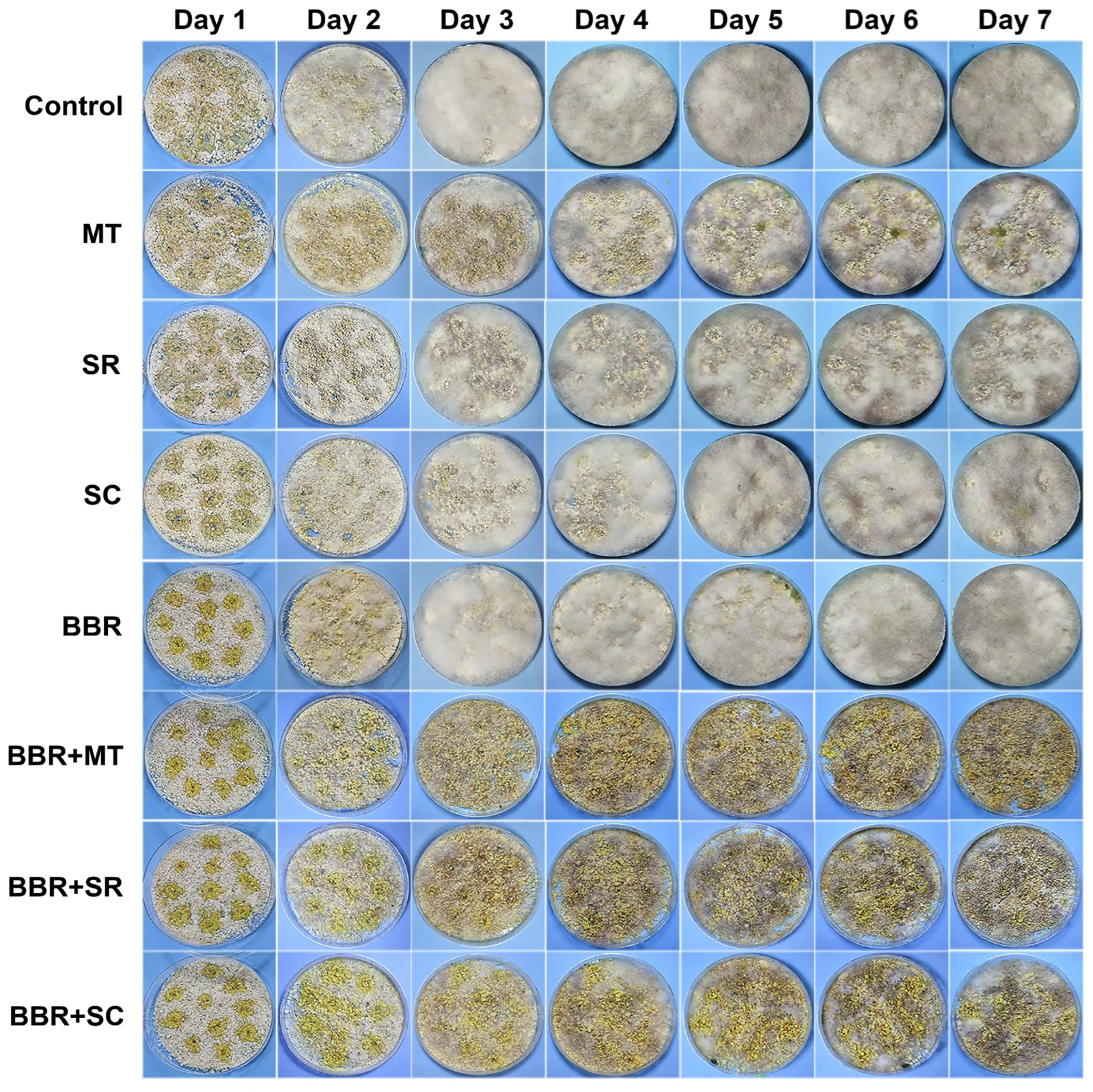
Inhibitory effects of MT, SR, SC, and BBR, either individually or in combination, on mycelial growth of *F. graminearum* strain PH-1 in pig feed.

Parallel experiments conducted in pig feed revealed a similar pattern of inhibition (Figure 2). Compared with the untreated control, *F. graminearum* strain PH-1 showed visibly reduced mycelial growth upon exposure to MT, SR, or SC alone, as well as to their respective QA-BBR combinations. Consistent with the results on PDA plates, BBR alone exhibited only weak inhibitory activity. Notably, visual assessment indicated that the three QA-BBR combinations tended to produce a more pronounced inhibitory effect on mycelial growth than the respective QA alone.

**Figure 3:**
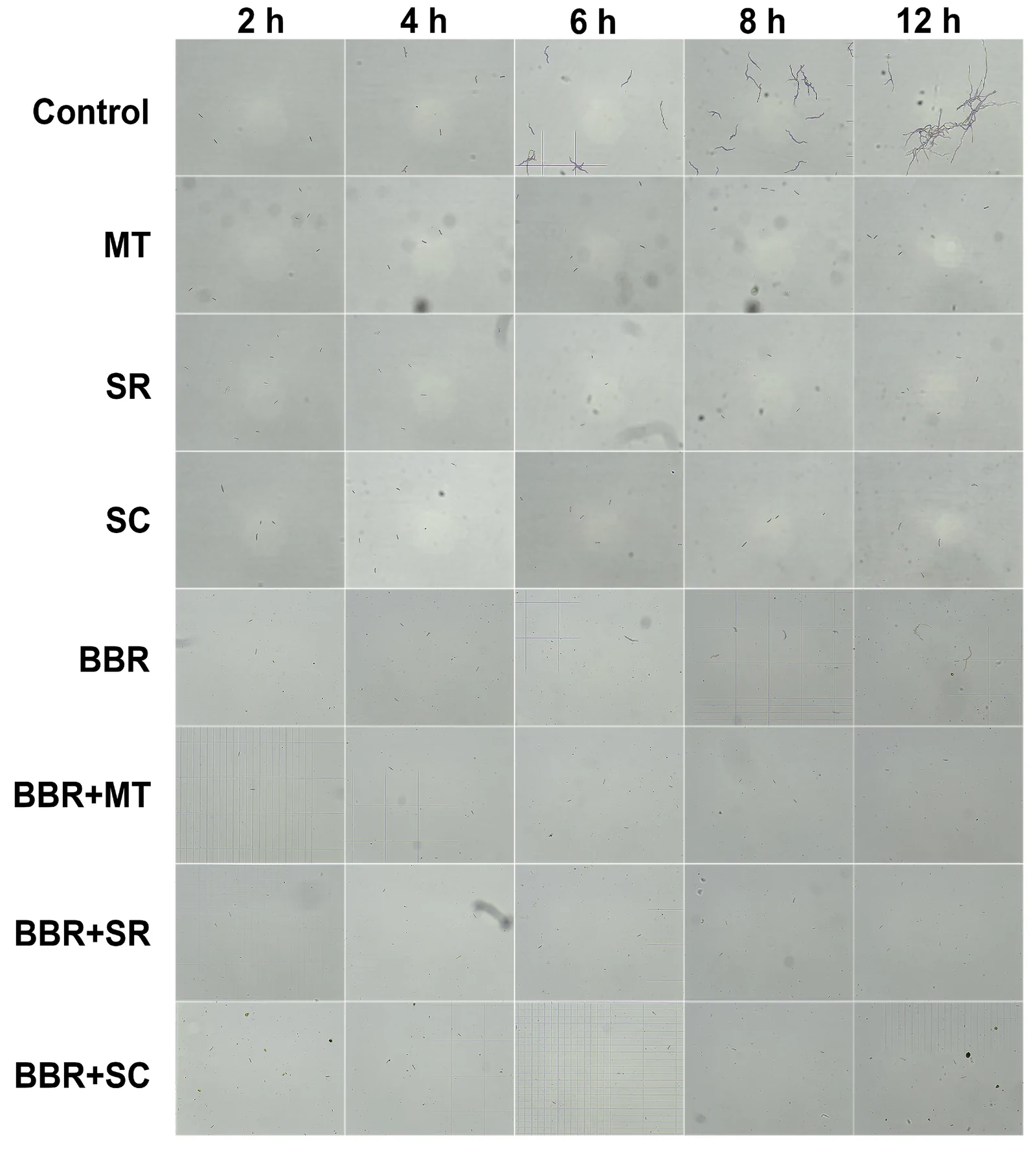
Inhibitory effects of MT, SR, SC, and BBR, either individually or in combination, on spore germination of *F. graminearum* strain PH-1.

As shown in Figure 3, all treatments inhibited spore germination of strain PH-1 compared with the untreated control, in which germination initiated at 4 h and form an extensive hyphal network by 12 h. Specifically, spore germination was completely inhibited within 12 h by treatment with MT (5 mg/mL), SR (4 mg/mL), or any combination of BBR (4 mg/mL) with MT (5 mg/mL), SR (4 mg/mL), or SC (4 mg/mL). In contrast, discernible germination was observed at 12 h after treatment with 4 mg/mL SC, and at 8 h after treatment with 4 mg/mL BBR.

**Figure 4:**
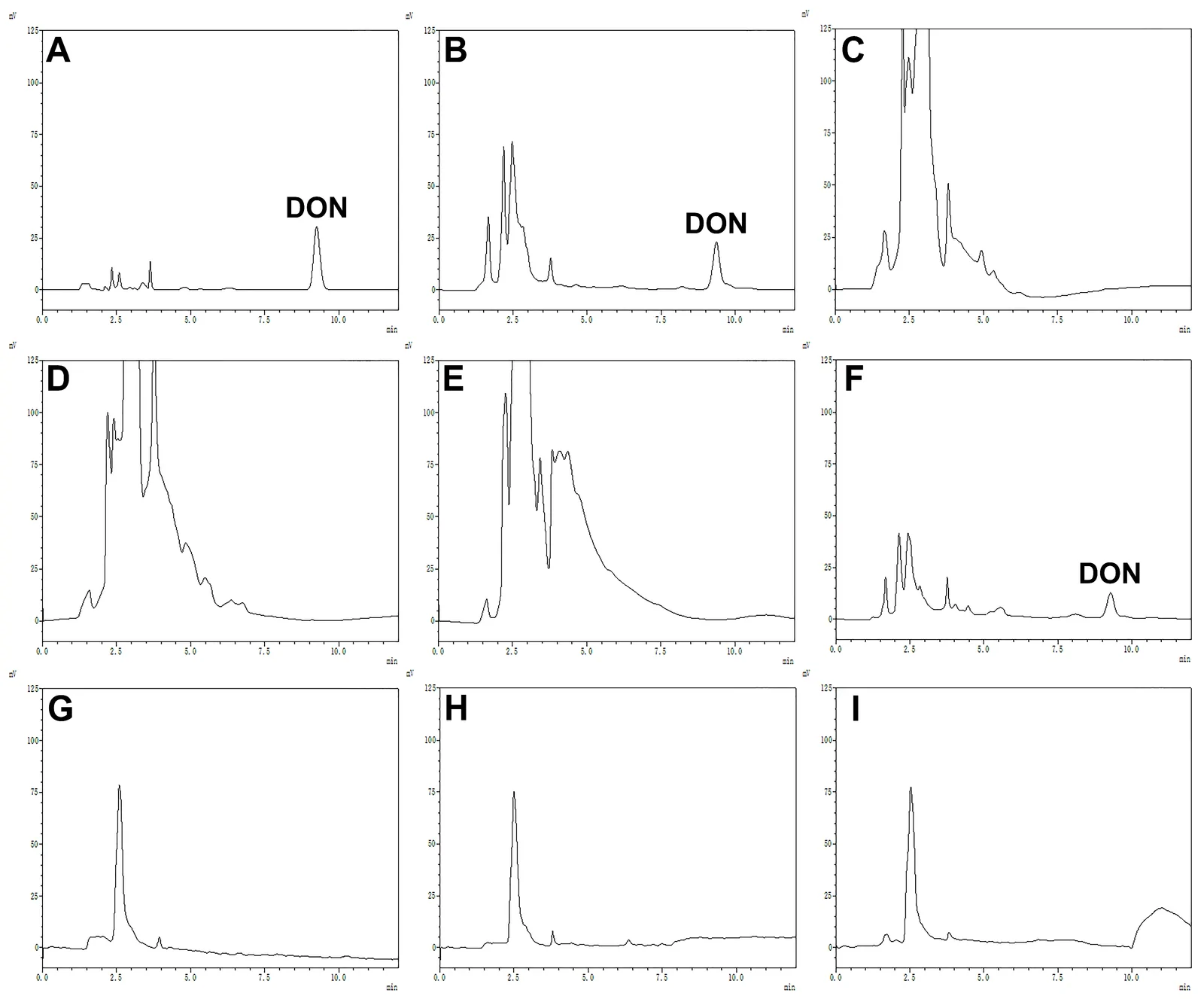
Representative high-performance liquid chromatography (HPLC) chromatograms of DON. (**A**) DON standard (20 μg/mL). Panels (**B**–**I**) show chromatograms of DON produced by *F. graminearum* strain PH-1 in TBI medium after 7 days of inoculated with different treatments: (**B**) Control; (**C**) MT (5 mg/mL); (**D**) SR (4 mg/mL); (**E**) SC (4 mg/mL); (**F**) BBR (4 mg/mL); (**G**) BBR (4 mg/mL)-MT (5 mg/mL); (**H**) BBR (4 mg/mL)-SR (4 mg/mL); (**I**) BBR (4 mg/mL)-SC (4 mg/mL).

The inhibitory effects of MT, SR, SC, and BBR on DON production by *F. graminearum* strain PH-1 grown in toxigenic biosynthesis induction (TBI) medium are shown in Figure 4. DON was detected at concentrations of 13.61 ± 3.38 μg/mL in untreated controls and 11.73 ± 1.23 μg/mL in BBR-treated samples. In contrast, the remaining samples, including those treated with MT, SR, or SC alone, as well as their respective combinations with BBR, showed no detectable levels of DON.

**Figure 5:**
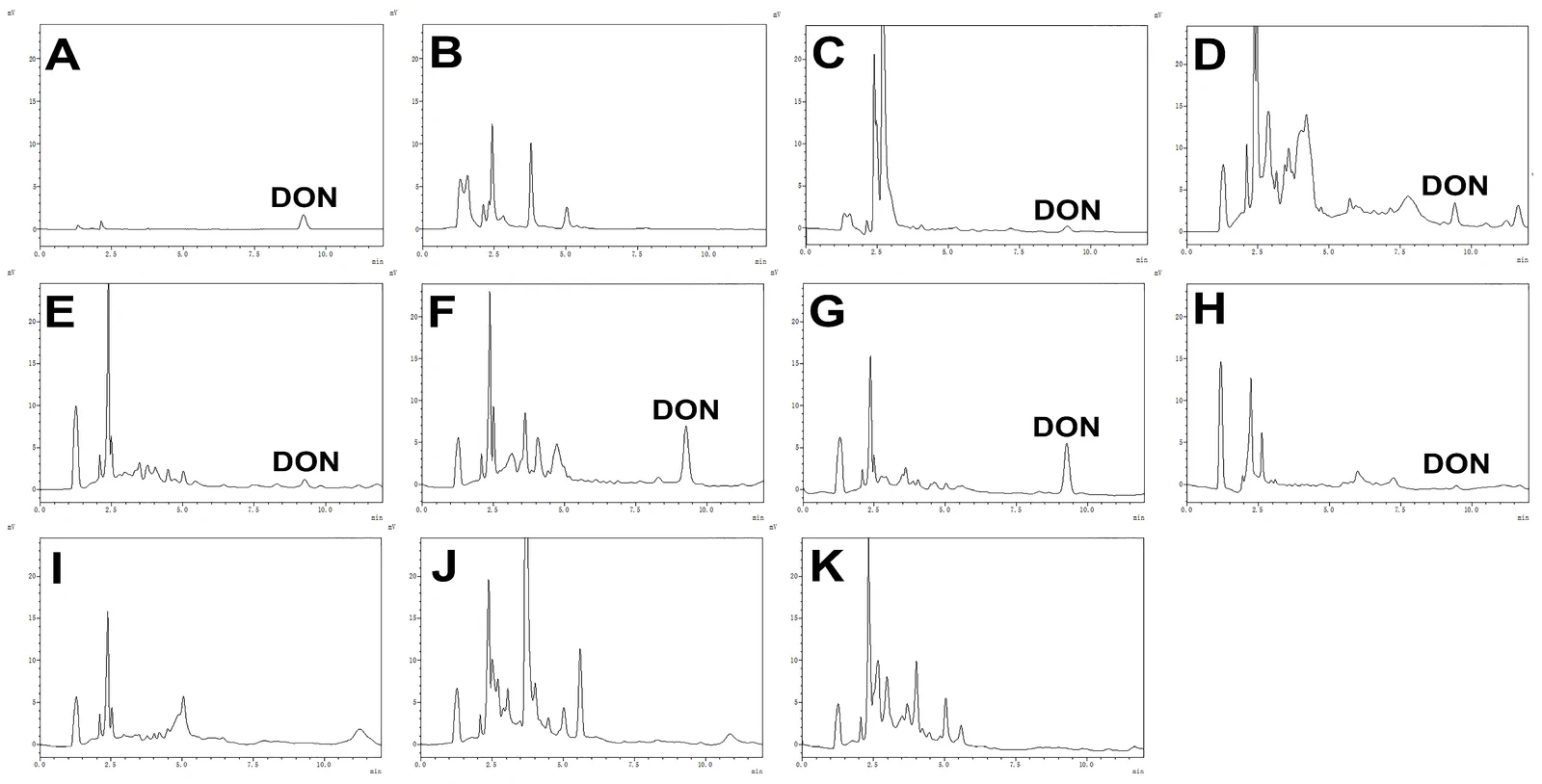
Representative HPLC chromatograms of DON. (**A**) DON standard (1 μg/mL); (**B**) blank pig feed; (**C**) blank feed spiked with DON (0.5 μg/g). Panels (**D**–**K**) show chromatograms of DON in pig feed inoculated with *F. graminearum* strain PH-1 and incubated for 7 days with different treatments: (**D**) Control; (**E**) MT (5 mg/g); (**F**) SR (4 mg/g); (**G**) SC (4 mg/g); (**H**) BBR (4 mg/g); (**I**) BBR (4 mg/g)-MT (5 mg/g); (**J**) BBR (4 mg/g)-SR (4 mg/g); (**K**) BBR (4 mg/g)-SC (4 mg/g).

Figure 5 shows the specificity of the HPLC method for determining DON in pig feed. The retention time of DON was approximately 9.25 min, with no endogenous interfering peaks observed in the corresponding region (Figure 5A,B). Table 1 summarizes the precision and accuracy of the HPLC method. Intra-day and inter-day relative standard deviations (RSDs) were below 9.47% and 7.91%, respectively, and recoveries ranged from 42.60% to 55.83%. For accurate quantification, a matrix-matched calibration curve (y = 5123.16x − 314.54) was constructed and demonstrated good linearity over the range of 0.5–10 μg/g (r = 0.9991). The instrumental limits of detection (LOD) and quantification (LOQ) were 0.25 μg/mL and 0.5 μg/mL, respectively. Accounting for the dilution factor during sample preparation, the method LOD and LOQ in feed matrix were 0.5 μg/g and 1.0 μg/g, respectively.

Using this validated HPLC method, we determined DON concentrations in pig feed. All feed samples were inoculated with *F. graminearum* strain PH-1 and incubated for 7 days. The treatment groups were supplemented with the aforementioned phytochemicals, while the control group contained no phytochemical. The corresponding results are presented in Figure 5D–K and Table 2. A significantly higher DON level was observed in feed samples supplemented with SR compared with the untreated control (*p* < 0.0077). Notably, no DON was detected in feed samples supplemented with QA-BBR combinations.

### 2.3. Differentially Expressed Genes (DEGs) and Their Functional Annotations in F. graminearum Strain PH-1 After Treatment with QAs Alone or in Combination with BBR

**Figure 6:**
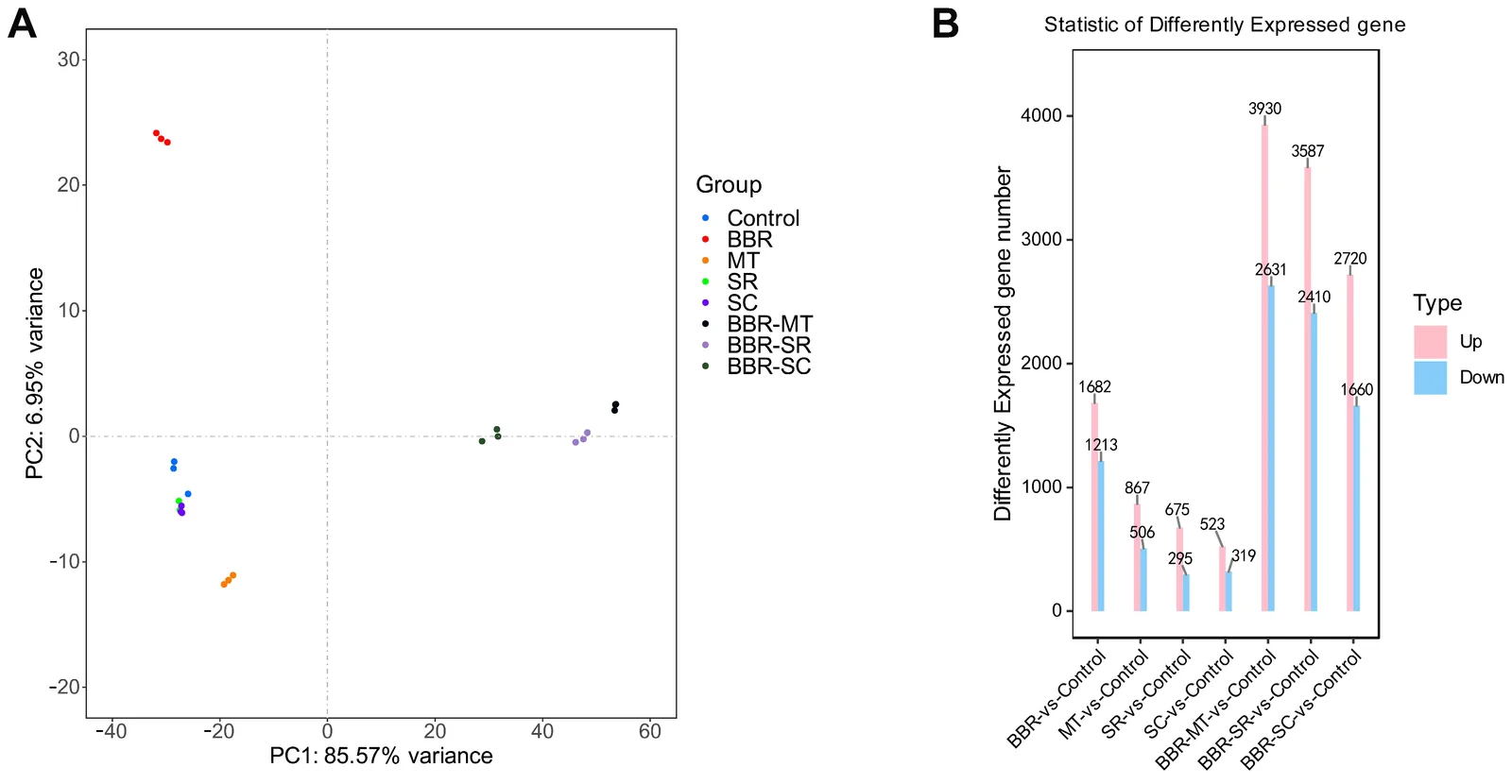
Transcriptome-wide variation and summary of DEGs across treatments. (**A**) Principal component analysis (PCA) of all samples. Each point represents a biological replicate. (**B**) Bar graph showing the counts of significantly upregulated (pink) and downregulated (blue) genes identified in each treatment group compared to the control (|log_2_FC| > 1, *q*-value < 0.05).

Principal component analysis (PCA) revealed high reproducibility among biological replicates within each group (Figure 6A). MT, SR, and SC treatments alone clustered tightly with the untreated control, despite inducing measurable phenotypic changes. In contrast, the BBR-treated groups (BBR alone or in combinations with MT, SR, or SC) exhibited distinct clustering separated from the untreated control. At the assessed time point, BBR alone triggered marked alterations in gene expression without concomitant phenotypic changes, whereas its combinations with these QAs (MT, SR, SC) potentiated both transcriptional and phenotypic responses. Differential gene expression profiles were consistent with this clustering pattern (Figure 6B). BBR alone affected the expression of 2895 genes (1682 upregulated; 1213 downregulated), whereas MT, SR, and SC induced modest transcriptomic changes (523–867 upregulated and 295–506 downregulated genes per treatment). Notably, the combination treatments (BBR-MT, BBR-SR, BBR-SC) produced a broad transcriptional response, yielding 4380–6561 DEGs. The scale of these changes was approximately threefold greater than that of BBR alone, with the BBR-MT combination showing the most pronounced effect.

**Figure 7:**
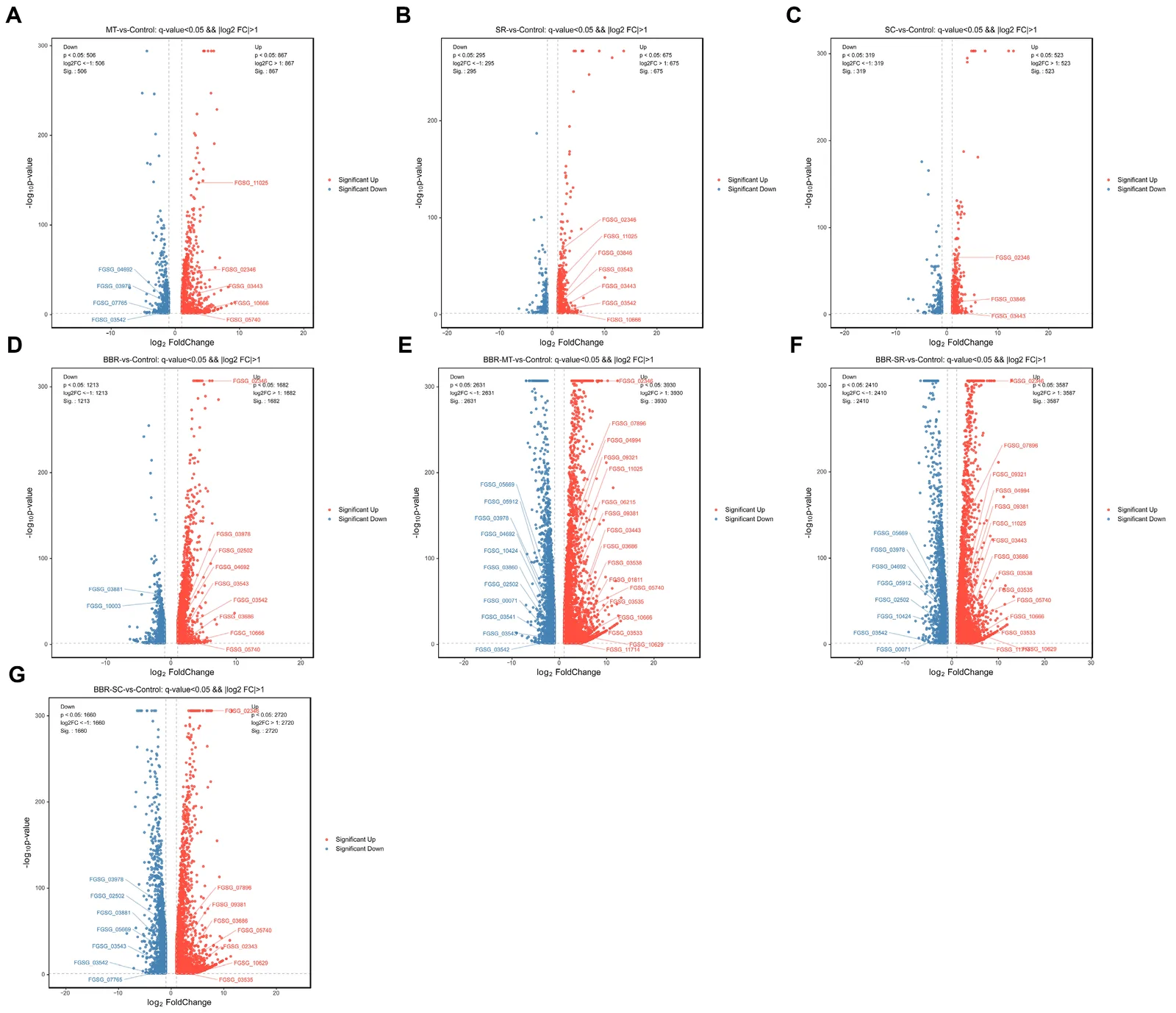
Transcriptomic changes in *F. graminearum* strain PH-1 treated with QAs alone or in combination with BBR. Volcano plots showing DEGs in each treatment compared to the control: (**A**) MT; (**B**) SR; (**C**) SC; (**D**) BBR; (**E**) BBR–MT; (**F**) BBR–SR; (**G**) BBR–SC. Red and blue dots represent significantly up- and down-regulated genes, respectively (|log_2_FC| > 1, *q*-value < 0.05).

**Figure 8:**
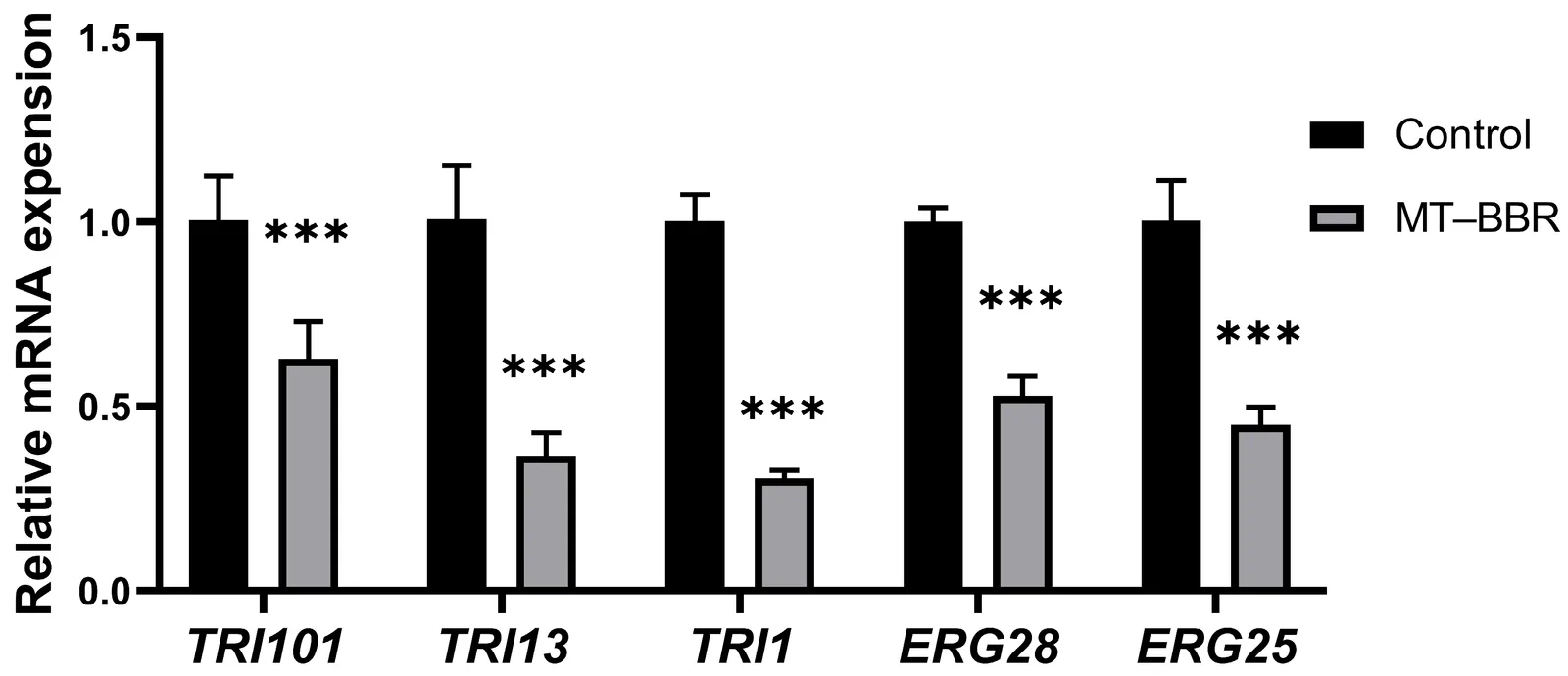
Effects of BBR–MT combination on the relative expression of genes involved in DON production and ergosterol biosynthesis, as determined by qRT-PCR ( *** *p* < 0.001). Data are presented as mean ± SD from three technical replicates.

Volcano plots revealed distinct expression patterns of DEGs across the seven treatment groups relative to the untreated control (Figure 7). The genes highlighted in the volcano plot insets are associated with DON production and ergosterol biosynthesis, belonging to the *TRI* gene cluster and the *ERG* genes, respectively (Supplementary Table S1). The combination treatments induced more pronounced transcriptional alterations in *TRI* and *ERG* genes compared to their respective single treatments, resulting in a greater number of DEGs within these gene sets. The quantitative real-time polymerase chain reaction (qRT-PCR) validated that MT combined with BBR markedly downregulated *TRI101 (*FGSG_04692*)*, *TRI13* (FGSG_03542), *TRI1* (FGSG_00071), *ERG25 (*FGSG_03978*)*, and *ERG28 (*FGSG_05669*)* (*p* < 0.001), consistent with the transcriptome profiles (Figure 8).

**Figure 9:**
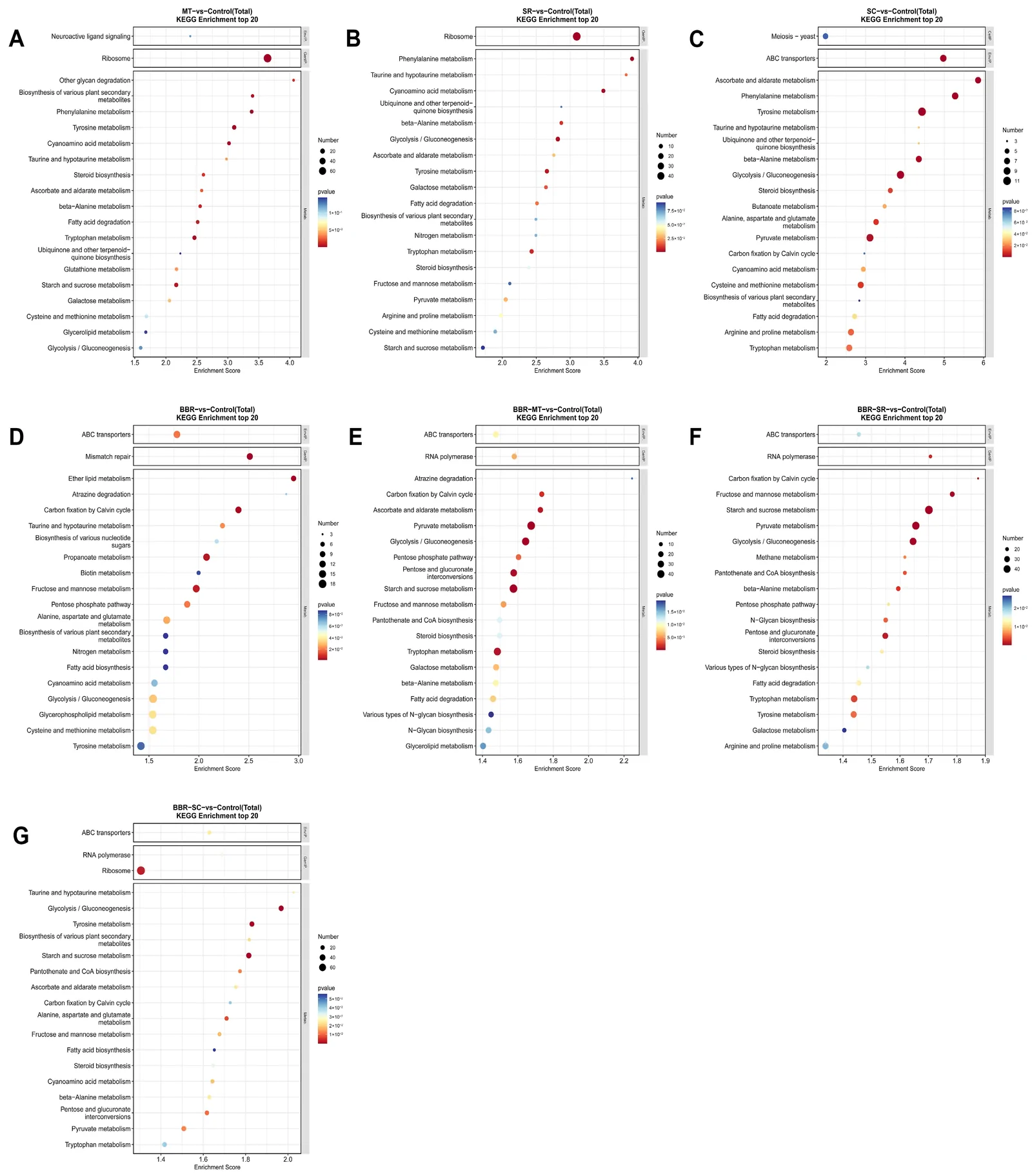
KEGG pathway enrichment analysis of DEGs (top 20 pathways). (**A**–**G**) show the comparisons of MT, SR, SC, BBR, BBR-MT, BBR-SR, and BBR-SC against the control, respectively. The *Y*-axis displays KEGG pathway terms (Level 3), and the *X*-axis indicates the enrichment score. Bubble color indicates the statistical significance based on *q* < 0.05 and |log_2_FC| > 1, with darker red denoting higher statistical significance; bubble size reflects number of DEGs annotated to each pathway.

Figure 9 shows the results of Kyoto Encyclopedia of Genes and Genomes (KEGG) pathway enrichment analysis. For the QAs-treated groups, DEGs were predominantly enriched in the ribosome pathway, ABC transporters, and amino acid metabolism pathways, particularly tyrosine and phenylalanine metabolism. In the BBR-treated group, by contrast, the major enriched pathways were ABC transporters, mismatch repair, ethyl hexanoate metabolism, and carbon fixation by the Calvin cycle. The enrichment profile in the combination-treatment groups shifted towards central carbon metabolism, encompassing glycolysis/gluconeogenesis, pyruvate metabolism, and the Calvin cycle, together with starch and sucrose metabolism, tyrosine metabolism, and the ribosome pathway. Notably, ergosterol biosynthesis-related DEGs were significantly enriched in steroid biosynthesis (a core pathway within the top 20 KEGG pathways) in the MT, SR, SC, BBR-MT, BBR-SC, and BBR-SR groups (Figure 9A–C, E–G).

**Figure 10:**
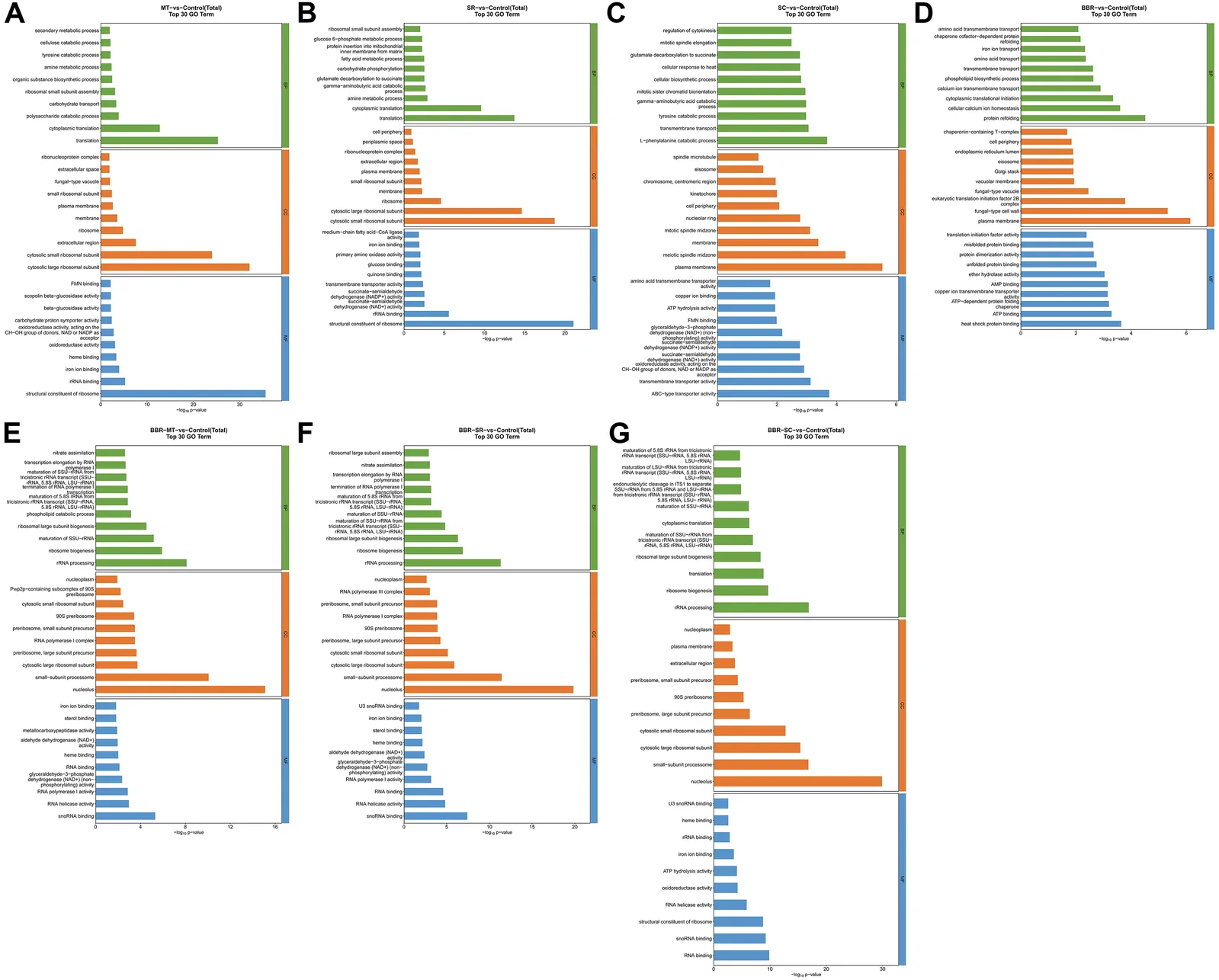
GO functional classification of DEGs. (**A**–**G**) represent the comparisons of MT, SR, SC, BBR, BBR-MT, BBR-SR, and BBR-SC against the control, respectively. The left *Y*-axis lists the top 30 significantly enriched GO terms, while the right *Y*-axis corresponds to the functional categories: BP, CC, and MF. The *X*-axis represents the enrichment significance (–log_10_ *p*-value).

Gene Ontology (GO) analysis revealed distinct functional enrichment across the treatment groups (Figure 10). Enriched GO terms were categorized into biological process (BP), cellular component (CC), and molecular function (MF). For the QAs-treated groups, DEGs were predominantly enriched in translation, cytoplasmic translation, L-phenylalanine catabolic process, and transmembrane transport (BP); cytosolic large ribosomal subunit, cytosolic small ribosomal subunit, plasma membrane, and meiotic spindle midzone (CC); and structural constituent of ribosome, rRNA binding, ABC-type transporter activity, and transmembrane transporter activity (MF). In the BBR-treated group, the major enriched GO terms were protein refolding and cellular calcium ion homeostasis (BP); plasma membrane and fungal-type cell wall (CC); and heat shock protein binding and ATP binding (MF). The enrichment profile in the combination-treatment groups shifted towards rRNA processing, ribosome biogenesis (BP); nucleolus, small-subunit processome (CC); and snoRNA binding and RNA helicase activity (MF).

## 3. Discussion

The frequent occurrence of DON in feedstuffs poses a persistent, multifaceted threat to global pig production, compromising animal health and performance, undermining feed safety, and constraining the sustainability of livestock systems. Against this backdrop, phytochemicals capable of inhibiting *F. graminearum* growth and DON production have attracted interest as potential in-feed anti-mycotoxigenic agents that complement conventional post-harvest control strategies. In the present study, MT, SR, and SC exhibited antifungal activity against *F. graminearum* and inhibitory effects on DON production, with particularly pronounced efficacy when applied in combination with BBR. These effects may be attributed to dysregulated expression of the *TRI* cluster genes and *ERG* biosynthesis genes, as well as to the differential expression of genes in the steroid biosynthesis (ko00100) and central carbon/lipid metabolism pathways. Overall, our findings establish proof-of-concept that combining MT, SR, or SC with BBR can inhibit DON production in laboratory culture media and pig feed, providing a foundation for future mechanistic dissection and the rational design of anti-mycotoxigenic interventions.

Among seven QAs and seven other phytochemicals tested, MT, SR, and SC were identified as active antifungal compounds against *F. graminearum*. Notably, combinations of these QAs with BBR exhibited enhanced antifungal activity. The antifungal activity of the three QAs (individually and in combination with BBR) was supported by observations of their inhibitory effects on mycelial growth, spore germination, and DON production. It is noteworthy that the antifungal efficacy of MT, SR, and SC appeared to be matrix-dependent. The three QAs at their MICs completely inhibited mycelial growth in potato dextrose broth (PDB), but were less effective on PDA plates and in pig feed. Specifically, hyphal regrowth was observed as early as 2 days after inoculation upon exposure to these QAs (Figure 1 and Figure 2). A similar pattern was observed for DON production: complete inhibition in liquid TBI medium (Figure 4), but virtually no inhibition in pig feed (Figure 5 and Table 2). Particularly, SR alone unexpectedly induced elevated DON levels in pig feed (Figure 5 and Table 2), revealing matrix-dependent divergent effects of SR on DON production. Insufficient or uneven exposure of *F. graminearum* to QAs presumably accounts for these observations. Indeed, unlike liquid cultures where components are uniformly distributed, solid matrices exhibit heterogeneous distributions of both fungal biomass and QAs. Local sublethal concentrations of antifungal compounds (e.g., SR) in pig feed may impose mild stress on *F. graminearum* and trigger signaling pathways that upregulate DON biosynthesis, as reported for other antifungal compounds [21]. Thus, thorough mixing is essential to ensure a homogeneous distribution of MT, SR, and SC in pig feed, thereby maximizing their antifungal activity against *F. graminearum* and their inhibitory effect on DON production. In recent years, liquid feeding has become increasingly popular in intensive pig farming due to its advantages in improving feed conversion ratio and pig health [5,6]. However, insufficient pipeline cleaning or incorrect water-to-feed ratios can promote fungal proliferation and DON accumulation [7], posing a significant dietary exposure risk. The three QAs evaluated here exhibited antifungal activity against *F. graminearum*, inhibitory effects on DON production, and desirable water solubility, supporting their potential relevance in liquid feeding systems, where homogeneous distribution can be more readily achieved. BBR, although exhibiting pronounced antifungal activity against certain pathogenic fungi (e.g., *Candida albicans*) [22], it had limited efficacy against *F. graminearum* strain PH-1 in the present study (Figure 1, Figure 2, Figure 3, Figure 4 and Figure 5 and Table 2). In stark contrast, the combination of BBR with the three QAs (MT, SR, and SC) exhibited potent antifungal efficacy against *F. graminearum*. This enhanced antifungal effect conferred nearly complete inhibition of mycelial growth, spore germination, and DON production across diverse matrices, including liquid cultures (PDB and TBI) and solid matrices (PDA and pig feed). Furthermore, this robust inhibition was maintained throughout the observation period, underscoring the theoretical and mechanistic value of this combinatorial strategy for controlling *F. graminearum* and its mycotoxins.

DON production serves as a direct and pivotal indicator for evaluating the efficacy of identified phytochemicals in mitigating DON contamination in pig feed. Accurate quantification of DON in pig feed is therefore essential. HPLC is arguably the most widely used method for this purpose, because it enables robust and reproducible quantification and is accessible even to routine analytical laboratories. However, HPLC coupled with ultraviolet detection often fails to effectively distinguish DON from co-extracted impurities in feed samples. Consequently, costly immunoaffinity column cleanup is typically required to ensure sufficient analytical specificity [23,24]. In this study, thin layer chromatography (TLC) was employed as a cost-effective alternative to immunoaffinity columns for purifying DON from feed extracts. Satisfactory analytical specificity was achieved (Figure 5). However, substantial DON loss occurred during the clean-up step, resulting in absolute recoveries ranging from 42.60% to 55.83% (Table 1). Advantageously, DON loss during the TLC clean-up step was consistent across all tested spiking concentrations, resulting in low absolute recovery but good precision. This consistent loss profile allows effective error compensation using matrix-matched calibration curve. Therefore, to ensure accurate quantification despite the low absolute recovery, a matrix-matched calibration curve was established by spiking blank feed samples with DON and subjecting these samples to the same preparation as incurred samples. With this calibration procedure, DON losses during the extraction and clean-up steps are equally accounted for in both calibration standards and test samples, and the final quantified values therefore reflect true DON concentrations in pig feed.

Mycelial growth of *F. graminearum* is closely linked to cellular ergosterol content [25,26]. Ergosterol biosynthesis is mediated by the fungal ergosterol biosynthetic branch of the KEGG steroid biosynthesis pathway (ko00100), a process catalyzed by enzymes encoded by the conserved *ERG* gene family [26,27]. Downregulation of key *ERG* genes (e.g., *ERG1*, *ERG3A*, *ERG3B*, *ERG4, ERG9, ERG24*) inhibits ergosterol biosynthesis in *F. graminearum* [26,27,28,29]. Interestingly, transcriptomic analysis revealed significant enrichment of the steroid biosynthesis (KEGG ko00100) pathway upon treatment with QAs and the BBR-QA combinations, with the majority of DEGs in this pathway showing upregulated. This upregulation appeared to reflect an ineffective compensatory response of *F. graminearum* to restore ergosterol homeostasis. Despite this transcriptional activation, mycelial growth remained inhibited (Figure 1 and Figure 2), suggesting that QAs and QA-BBR combinations may impair ergosterol biosynthesis at the post-transcriptional or enzymatic level, thereby preventing the conversion of enhanced gene expression into functional sterols. Such a mismatch between excessive transcription and blocked biosynthesis may lead to severe depletion of cellular energy reserves, as supported by the concomitant downregulation of DEGs involved in glycolysis/gluconeogenesis, glycerolipid, glycerophospholipid, and pyruvate metabolism pathways. This energy depletion may in turn contribute to the inhibition of mycelial growth.

The biosynthesis of DON in *F. graminearum* is catalyzed by enzymes encoded by the *TRI* cluster genes [30]. *TRI1* and *TRI11*, which encode the cytochrome P450 monooxygenase and the C-15 hydroxylase, respectively, are essential structural genes for late-stage DON biosynthesis. Their downregulation leads to a substantial reduction in DON production [31,32]. Transcriptomic analysis revealed that *TRI1* was significantly downregulated upon the exposure of *F. graminearum* to MT-BBR and SR-BBR combinations, whereas *TRI11* was significantly downregulated in response to SC-BBR treatment (Supplementary Table S1). The coordinated inhibition of these late-stage biosynthetic genes offers a plausible molecular explanation for the observed reduction in DON production (Figure 4 and Figure 5).

Transcriptomic profiling further revealed the molecular basis of the enhanced antifungal and antimycotoxigenic effects observed in the combined treatment groups. PCA clustering revealed distinct transcriptional response patterns between individual and combined treatments, with combination groups clearly separating from both the control and individual treatment clusters (Figure 6A). Consistent with this, combination treatments induced markedly broader transcriptomic perturbations, with DEG numbers ranging from 4380 to 6561, approximately threefold greater than induced by BBR alone (Figure 6B). These results suggest that the transcriptional remodeling driven by QA–BBR combinations may contribute to the observed enhancement of antifungal and antimycotoxigenic effects. KEGG enrichment analysis revealed treatment-specific metabolic perturbations. The three QAs primarily perturbed ribosome function, ABC transporters and aromatic amino acid metabolism, whereas BBR interfered with ABC transporters, mismatch repair, ethyl hexanoate metabolism, and carbon fixation by the Calvin cycle. In contrast, QA–BBR combinations exhibited a shift in transcriptional perturbations toward central carbon metabolism (glycolysis/gluconeogenesis and pyruvate metabolism), as well as starch and sucrose metabolism, while retaining disturbances in ribosome and tyrosine metabolism. Additionally, the steroid biosynthesis pathway (within the top 20 enriched KEGG pathways) was significantly enriched in both QA-treated groups and QA–BBR combination groups. By concurrently perturbing energy metabolism, translational processes and steroid biogenesis, QA–BBR combinations may impose greater metabolic stress on *F. graminearum*, thereby inhibiting fungal growth and DON production more effectively than individual treatments.

The marked enrichment of DEGs in glycolysis and pyruvate metabolism in combination-treated groups warrants careful attention. These central carbon metabolism pathways collectively form a critical integrative node linking secondary metabolite biosynthesis and cellular redox homeostasis. Flux through glycolysis and pyruvate metabolism governs the availability of acetyl-CoA, a precursor for DON biosynthesis [33,34]. Meanwhile, glucose-6-phosphate (G6P) acts as a branching point metabolite shared by glycolysis and the pentose phosphate pathway, and fluctuations in global central carbon flux redirect G6P to modulate carbon flux into the NADPH-generating pentose phosphate pathway [34,35]. Upon combined treatment, concomitant downregulation of DEGs involved in glycolysis and pyruvate metabolism may therefore exert dual effects: it limits acetyl-CoA availability to hinder DON biosynthesis and alters G6P partitioning, which in turn potentially reduces cellular NADPH pools required to sustain redox homeostasis. Importantly, although our metabolic inference links perturbation of central carbon metabolism to altered cellular redox status, the upstream signal transduction cascades triggering fungal antioxidant defense responses remain uncharacterized based on current data. Future experiments including reactive oxygen species quantification, antioxidant enzyme activity assays and redox-sensitive biosensor detection are essential to validate this hypothesis.

It should be noted that the antifungal efficacy of the three QAs (MT, SR, SC) and their combinations with BBR was observed only at relatively high concentrations. Such concentration levels may raise concerns regarding safety and cost-effectiveness, limiting their immediate feasibility for practical field applications. Accordingly, application-oriented optimization strategies are warranted to reduce current effective concentrations. One strategy to address this is to target scenario-specific deployment, such as liquid feeding systems. In these application scenarios, MT, SR, SC, and BBR exhibit enhanced dispersibility, enabling equivalent efficacy at substantially reduced concentrations. Complementing this, co-application of QAs or QA-BBR combinations with organic acids (e.g., propionic acid, sorbic acid) or phenolic essential oils (e.g., thymol, carvacrol) may be an effective strategy to reduce the MIC against *F. graminearum*. Beyond chemical synergies, incorporating commercial GRAS yeasts (*Saccharomyces cerevisiae*) or bacilli (*Bacillus subtilis*) in liquid feeding systems may represent a complementary biocontrol strategy. In this microbial consortium, sub-MIC concentrations of QAs or QA-BBR combinations may be sufficient to inhibit the mycelial growth and DON production of *F. graminearum*, as this pathogen is already disadvantaged by nutrient competition and acidification.

## 4. Conclusions

In summary, MT, SR and SC exhibited antifungal activity against *F. graminearum* and inhibitory effects on DON production in liquid culture media. However, these QAs alone did not produce comparable effects in pig feed, indicating matrix-dependent characteristics. Notably, QA-BBR combinations markedly enhanced their antifungal activity and inhibitory effects on DON production in laboratory culture media and pig feed, albeit at relatively high concentrations. Further investigation into the practical application and safety of these combinations at the concentrations employed herein is warranted.

## 5. Materials and Methods

### 5.1. Chemicals, Fungal Strain and Pig Feed

MT, oxymatrine (OMT, CAS 16837-52-8), oxysophocarpine (OSC, CAS 26904-64-3), cytisine (CYT, CAS 485-35-8), (-)-sparteine sulfate pentahydrate (SPT-S·5H_2_O, CAS 6160-12-9), gallic acid (GA, CAS 149-91-7), rosmarinic acid (RA, CAS 20283-92-5), paeoniflorin (PF, CAS 23180-57-6), chlorogenic acid (CGA, CAS 327-97-9), palmatine (PAL, CAS 3486-67-7), and pulsatilla saponin B4 (PSB4, CAS 129741-57-7), all with purities exceeding 98.0%, were purchased from Yuanye Bio-Technology Co., Ltd. (Shanghai, China). SR, SC, and BBR, all with purities exceeding 98.0%, were obtained from Macklin Biochemical Technology Co., Ltd. (Shanghai, China). Analytical standard of DON (purity > 95.0%) was purchased from Shanghai J&K Scientific Ltd. (Shanghai, China). HPLC-grade acetonitrile was supplied by Merck KGaA (Darmstadt, Germany), while analytical-grade acetonitrile, methanol, ethyl acetate, *n*-hexane, isopropanol, and dichloromethane were obtained from Sinopharm Chemical Reagent Co., Ltd. (Shanghai, China). Ultrapure water (resistivity: 18.25 MΩ·cm) was used throughout all experimental procedures. Individual stock solutions of MT, SR, SC, OMT, OSC, CYT, and SPT-S·5H_2_O were prepared in ultrapure water at a concentration of 20 mg/mL for MT and 16 mg/mL for the remaining six compounds. Stock solutions of the remaining seven phytochemicals were prepared individually in ultrapure water at their respective maximum soluble concentrations. These concentrations were determined as the highest concentration at which no precipitate was observed after sonication and equilibration at room temperature (Supplementary Table S2). Subsequently, all aqueous solutions (including the stock solutions and their dilutions) were sterilized by filtration through 0.22 μm membranes (Jinteng Laboratory Equipment Co., Ltd., Tianjin, China) and used immediately for antifungal assays. Additionally, a stock solution of DON was prepared in acetonitrile at 2.5 mg/mL and stored at −20 °C.

The *F. graminearum* strain PH-1 (standard wild-type, NRRL31084) was a gift from Prof. Huawei Zheng (Fujian Agriculture and Forestry University, Fuzhou, China). The strain was routinely cultured on PDA (Hope Bio-technology Co., Ltd., Qingdao, China) plates at 25 °C. For long-term preservation, it was cryopreserved at −80 °C in sterile cryotubes containing 20% (*v*/*v*) glycerol, using mycelial plugs from 3-day-old PDA cultures. The spore suspension of strain PH-1 was prepared as follows: A 6 mm-diameter mycelial plug was taken from the edge of a 3-day-old PDA colony using a sterile cork borer. Five such plugs were transferred into a conical flask containing 50 mL of carboxymethyl cellulose (CMC) liquid medium (Ruichu Biotechnology Co., Ltd., Shanghai, China). The flask was incubated at 28 °C with shaking at 180 rpm for 5 days to induce sporulation. After incubation, the culture was filtered through four layers of sterile cheesecloth to remove mycelial debris. The filtrate was centrifuged at 3000 rpm for 10 min to pellet the spores. The pellet was resuspended in an appropriate volume of sterile water, and the spore concentration was determined using a hemocytometer under an optical microscope. The suspension was stored at 4 °C and used within 2 h.

The pig feed was obtained from Beijing Chia Tai Feedmill Co., Ltd. (Beijing, China). Prior to the experiment, this feed sample was confirmed to be free of detectable DON (below the LOQ) by HPLC analysis.

### 5.2. Screening of Antifungal Phytochemicals and Their Potentiating Agents Against F. graminearum

The susceptibility of strain PH-1 to seven QAs and seven additional phytochemicals was determined by a modified microdilution method, as described previously [36,37]. Briefly, each well of a 96-well plate was loaded with 100 μL of aqueous solutions containing two-fold serial dilutions of each phytochemical. The detailed concentration ranges are provided in Supplementary Table S3. A well containing no phytochemical served as the negative control. The spore suspension was diluted with double-strength PDB (Hope Bio-technology Co., Ltd., Qingdao, China) to 1.0 × 10^6^ spores/mL, and then 100 μL of the resulting dilution was added to each well. The 96-well plates were covered and incubated at 25 °C for 72 h. The MIC was defined as the lowest concentration of the phytochemical that completely inhibited visible growth of strain PH-1. After MIC determination, 50 µL aliquots were withdrawn from the well corresponding to the MIC and from 2 to 3 subsequent wells with increased concentrations (all exhibiting visual clarity). These aliquots were evenly spread onto phytochemical-free PDA plates, and incubated at 25 °C for 72 h to evaluate mycelial growth. The MFC was defined as the lowest concentration that completely prevented visible fungal growth on the plates after incubation. The phytochemicals exhibiting antifungal activity were screened based on their MIC and MFC values.

To evaluate combinatorial antifungal effects, the screened antifungal phytochemicals were pairwise combined with each of the inactive phytochemicals. The resulting combinations were then incubated with strain PH-1 at 25 °C for 72 h to determine mycelial growth. In the combined treatment system, the concentration of the antifungal phytochemical was set at sub-inhibitory levels (¼× to ½× its individual MIC), while the inactive phytochemical was tested at its maximum soluble concentrations. A negative (growth) control, which contained strain PH-1 without any phytochemicals, was included in each experiment. An enhanced effect was attributed to the inactive phytochemical when its combination with an antifungal partner at sub-inhibitory concentrations led to complete inhibition of strain PH-1 growth, as indicated by a clear well.

### 5.3. Evaluation of the Antifungal Efficacy of Phytochemicals Alone and Incombination

#### 5.3.1. Mycelial Growth Inhibition Assay

The mycelial growth inhibition assay was conducted in two distinct experimental systems: PDA plates and pig feed matrix.

For the assay on PDA plate, the mycelial growth of strain PH-1 was evaluated after the fungus was treated with the screened antifungal phytochemicals (MT, SR, SC), individually or in combination with their potentiating agent (BBR). The PDA medium was amended with the respective phytochemicals, sterilized, and poured into sterile plates. The final concentrations of MT, SR, SC, and BBR in the PDA medium are listed in Supplementary Table S4. A 6 mm diameter mycelial plug was excised from the edge of a 3-day-old PDA colony and then inoculated at the center of each PDA plate. A negative control, which contained strain PH-1 without any phytochemicals, was included. The plates were incubated at 28 °C for 3 days under normal light/dark cycles. Three independent biological replicates were performed for each treatment.

For the assay in pig feed, the experiment followed a similar protocol. Briefly, 5 g of sterilized pig feed was evenly spread to form a thin layer in a 9 cm disposable Petri dish. Aliquots of the stock solutions were added to the feed to achieve the final theoretical concentrations specified in Supplementary Table S4. After the addition of the phytochemicals, the feed in each Petri dish was air-dried to remove excess moisture. Subsequently, the dried feed-phytochemical mixtures were inoculated with 1 mL of a spore suspension (1.0 × 10^6^ spores/mL). To ensure even distribution, both the phytochemical stock solutions and the spore suspension were dispensed as ten droplets at evenly spaced points across the Petri dish. A negative control (PH-1 only, without phytochemicals) was included. Each treatment was performed in five replicates. All Petri dishes were incubated under controlled conditions (28 °C, 80% relative humidity) for 7 days under normal light/dark cycles. Mold growth in these feed samples was assessed by daily visual inspection.

#### 5.3.2. Spore Germination Inhibition Assay

The spore germination of strain PH-1 was evaluated in 30 mL of PDB medium after the fungus was treated with MT, SR, SC, either individually or in combination with BBR. The PDB medium was amended with the respective phytochemicals, sterilized, and dispensed into sterile conical flasks. The final concentrations of MT, SR, SC, and BBR in the PDB medium are listed in Supplementary Table S4. Subsequently, 1 mL of a spore suspension (1 × 10^6^ spores/mL) was inoculated into each flask. A negative control (PH-1 only, without phytochemicals) was included. Each treatment was performed in three replicates. The flasks were incubated at 28 °C in a shaking incubator at 180 rpm. After 2, 4, 6, 8, and 12 h of incubation, 10 μL aliquots were withdrawn from each treatment, placed on a hemocytometer, and examined microscopically to evaluate spore germination.

#### 5.3.3. DON Production Inhibition Assay

The DON production inhibition assay was conducted in two distinct experimental systems: TBI medium and pig feed.

For the assay in TBI medium, the DON production of strain PH-1 was evaluated after strain PH-1 was treated with MT, SR, SC, either individually or in combination with BBR. The TBI medium (30 mL) was amended with the respective phytochemicals at the concentrations specified in Supplementary Table S4, sterilized, and dispensed into sterile conical flasks. Subsequently, 1 mL of a spore suspension (1 × 10^6^ spores/mL) was inoculated into each flask. The flasks were incubated at 28 °C in a shaking incubator at 180 rpm. A negative control (PH-1 only, without phytochemicals) was included. Each treatment was performed in three replicates. After 7 days of incubation, the culture medium from each flask was sequentially filtered through a 0.5 mm mesh (Haining Guodian Taoyuan Medical and Chemical Instrument Factory, Jiaxing, China) and a 0.22 μm filter (Jinteng Laboratory Equipment, Tianjin, China). A 20 μL aliquot of the resulting filtrate was then injected for HPLC analysis.

The analytical conditions were as follows: HPLC system, Shimadzu LC-16 HPLC system (Shimadzu Corporation, Kyoto, Japan); chromatographic column, Shim-pack GIST C_18_ column (4.6 × 250 mm, 5 μm; Shimadzu, Kyoto, Japan); column temperature, 35 °C; mobile phase, acetonitrile–water (11:89, *v*/*v*); flow rate, 1 mL/min; ultraviolet detector, 220 nm. Quantification was performed using a standard curve, which was prepared by diluting DON standard in the mobile phase to concentrations ranging from 0.1 to 10 μg/mL.

The inhibitory effects of MT, SR, SC, and BBR on DON production by strain PH-1 were also evaluated in the pig feed. The procedures for phytochemical addition, fungal inoculation, and incubation, as well as the arrangement of treatment groups and replicates, were identical to those used in the protocol for the mycelial growth inhibition assay in the pig feed. After 7 days of incubation, the DON concentration in the feed samples was analyzed using an HPLC method.

The sample pretreatment was performed as follows: individual, intact feed samples (approximately 5 g) were dried in an oven at 60 °C for 24 h, and then finely ground to a fine powder. The ground sample was quantitatively transferred to a 50 mL centrifuge tube, and 10 mL of 84% (*v*/*v*) acetonitrile–water solution was added. The mixture was vortexed for 2 min and centrifuged at 11,000 rpm for 5 min. The resulting supernatant was transferred to a new 50 mL centrifuge tube. The extraction was repeated once with an additional 10 mL of the same acetonitrile–water solution. An aliquot (one-fifth) of the combined supernatants was evaporated to dryness under a gentle stream of nitrogen at 65 °C. The dried residue was reconstituted in 150 μL of acetonitrile. Lipids in the dried residue were removed by extracting with 1 mL of *n*-hexane saturated with acetonitrile. Further purification of the defatted sample was performed by TLC using a silica gel 60 GF_254_ plate (Merck KGaA, Darmstadt, Germany). The stock solution of DON (2.5 mg/mL, 20 μL) and the enriched feed extracts (75 μL) were spotted separately onto the TLC plate. The TLC plate was developed in a pre-saturated developing chamber at room temperature with dichloromethane-isopropanol (9:1, *v*/*v*) to a distance of 8 cm, then air-dried and immediately visualized under 254 nm UV light. The bands corresponding to DON in the sample lanes (feed extracts) were identified by aligning their horizontal positions with that of the co-spotted DON standard. The silica gel zones containing DON were carefully marked and scraped from the plate. DON was extracted once from the collected silica gel with 1 mL of the mobile phase. After brief centrifugation, the supernatant was filtered through a 0.22 μm filter (Jinteng Laboratory Equipment, Tianjin, China), and the filtrate was analyzed by HPLC under the previously described analytical conditions.

The analytical method was validated for its specificity, linearity, accuracy, precision, and sensitivity. The specificity was confirmed by comparing the chromatograms of twenty blank feed samples with those of spiked samples. The concentrations of DON in feed samples were quantified using a matrix-matched calibration curve. The curve was prepared by spiking blank feed samples with DON at 0.5, 1, 2, 5, 10 μg/g (five replicates per concentration) and subjecting them to the same pretreatment and analytical procedures as the incurred samples. This calibration procedure enables compensation for consistent DON losses occurring during sample extraction and clean-up. The linearity of the calibration curve was assessed by the correlation coefficient (r). The accuracy and precision were evaluated by analyzing the spiked samples at 0.5, 2, 10 μg/g (5 replicates per concentration) over three consecutive days, with results reported as the recoveries and RSD. The sensitivity was evaluated by the instrumental LOD and LOQ. These were determined by analyzing blank feed extracts spiked with DON at five concentrations (0.05–10 μg/mL), with LOD and LOQ defined as the lowest concentrations yielding signal-to-noise ratios of 3 and 10, respectively.

### 5.4. Transcriptome Sequencing

Transcriptome sequencing was performed on *F. graminearum* strain PH-1 after treatment with MT, SR, SC, and BBR. The sequencing service was provided by Shanghai OE Biotech Co., Ltd. (Shanghai, China). A total of twenty-four samples were sequenced, with three biological replicates for each of the following eight groups: untreated control; MT (5 mg/mL); SR, SC, and BBR (each at 4 mg/mL); and the three combinations of BBR (4 mg/mL) with MT (5 mg/mL), SR (4 mg/mL), and SC (4 mg/mL), respectively. The samples for sequencing were prepared as follows. First, 50 mL of Yeast Extract Peptone Dextrose (YEPD) medium (Coolaber Science & Technology Co., Ltd., Beijing, China) was prepared, sterilized, and dispensed into sterile 150 mL conical flasks. Each flask was then inoculated with 1 mL of spore suspension (1 × 10^6^ spores/mL) and incubated in a shaking incubator at 28 °C and 180 rpm for 24 h. After this pre-culture period, the flasks were divided into the eight groups. The respective phytochemicals were added to the treatment groups at the indicated concentrations, while the untreated control group received an equal volume of sterile water. All groups were then incubated under the same conditions for an additional 24 h. Thereafter, the mycelia from each flask were harvested by filtration through four layers of sterile gauze. After three washes with sterile water, the mycelia were transferred into cryovials, flash-frozen in liquid nitrogen, and stored at −80 °C. Total RNA was extracted using TRIzol reagent (Invitrogen Co., Carlsbad, CA, USA), followed by assessment of RNA purity and integrity using an Agilent 2100 Bioanalyzer (Agilent Technologies, Santa Clara, CA, USA). Transcriptome libraries were constructed using the VAHTS Universal V10 RNA-seq Library Prep Kit (Premixed Version) (Vazyme Biotech Co., Ltd., Nanjing, China) and were subsequently sequenced on an Illumina Novaseq 6000 platform (Illumina, Inc., San Diego, CA, USA). Raw reads were processed with Fastp (version 0.20.1) to remove adapter sequences and low-quality reads [38]. Clean reads were aligned to the *F. graminearum* PH-1 reference genome (assembly accession ID: ASM24013v3) using HISAT2 (version 2.1.0) [39]. Gene expression levels were quantified as Fragments Per Kilobase Million (FPKM) values, and read counts per gene were obtained using HTSeq-count (version 0.11.2) [40]. Differential expression analysis was carried out with DESeq2 (version 1.22.2) [41], and genes meeting the thresholds of *q* < 0.05 and |fold change| > 2 were defined as DEGs. Functional enrichment analyses of DEGs, including GO terms and KEGG pathways, were performed based on the hypergeometric distribution to identify significantly enriched terms [42,43]. Gene Set Enrichment Analysis (GSEA) was performed using the GSEA software (version 4.3.0) [44]. In this analysis, genes were ranked based on their differential expression between two or more experimental conditions. Predefined gene sets were then evaluated for significant enrichment at either the top or bottom of the ranked gene list.

To validate the transcriptomic data, qRT-PCR was performed to determine the expression levels of five DEGs in *F. graminearum* strain PH-1 after treatment with MT-BBR combinations. The five DEGs comprised three associated with DON biosynthesis (*TRI101*, *TRI13*, and *TRI1*) and two involved in ergosterol biosynthesis (*ERG25* and *ERG28*). Total RNA was extracted from each sample using a SteadyPureRNA Extraction Kit (Accurate Biotechnology (Hunan) Co., Ltd., Changsha, China). Then, cDNA was synthesized using Evo M-MLV Reverse Transcription Mix Kit from the same manufacturer. qRT-PCR was performed using the NovoStart Universal Fast SYBR qPCR SuperMix (Novoprotein Biotechnology Co., Ltd., Suzhou, China) on a QuantStudio 1 StepOne^TM^ plus Real-Time PCR System (Thermo Fisher Scientific Inc., Waltham, MA, USA). The qRT-PCR primers were designed using Primer-BLAST (http://www.ncbi.nlm.nih.gov/tools/primer-blast) (accessed on 12 December 2025). All primers were synthesized by Sangon Biotech Co., Ltd. (Shanghai, China), and their sequences are listed in Supplementary Table S5. The 20 μL qRT-PCR reaction mixture consisted of 10 μL of 2×NovoStart^®^Universal Fast SYBR qPCR SuperMix, 0.5 μL of each primer, 1 μL of cDNA template, and 8 μL of RNase free water. The qRT-PCR amplification was performed under the following thermal cycling conditions: initial denaturation at 95 °C for 30 s, followed by 40 cycles of 95 °C for 5 s and 60 °C for 15 s. A melt curve analysis was subsequently conducted with incubation at 95 °C for 15 s, 60 °C for 1 min, and a continuous temperature ramp from 60 °C to 95 °C with constant fluorescence acquisition. Representative amplification curves for each target gene are provided in Supplementary Material (Figure S1). The relative expression levels of the target genes were calculated using the 2^−ΔΔCt^ method, with the *FgActin* gene as the internal control. Three independent biological replicates were included for each sample, with each replicate subjected to three technical parallel measurements.

### 5.5. Data Analysis

All quantitative data, including the DON concentrations in TBI medium and feed samples, as well as the RE and RSD, were presented as mean ± standard deviation (SD). Statistical analyses were performed using Prism software (version 8.0.2, GraphPad Software, Inc., San Diego, CA, USA). Differences among treatment groups were analyzed using a two-tailed Student’s *t*-test. Significance levels are indicated as follows: * *p* < 0.05, ** *p* < 0.01, *** *p* < 0.001.

## Figures and Tables

**Table 1:** Precision and accuracy of the HPLC method for determining of DON in pig feed.

Spiked Concentration (μg/g)	Recovery (%) ^a^	Intra-Day RSD (%)	Inter-Day RSD (%)
10	50.08 ± 0.03	6.91	6.03
	48.17 ± 0.02	4.69	
	46.42 ± 0.02	4.36	
2	49.08 ± 0.04	8.65	6.44
	50.14 ± 0.03	6.22	
	48.92 ± 0.03	5.25	
0.5	49.94 ± 0.05	9.06	7.91
	49.59 ± 0.05	9.47	
	49.34 ± 0.03	6.76	

^a^ Mean ± SD, *n* = 5.

**Table 2:** DON concentrations in pig feed after inoculation with *F. graminearum* strain PH-1 and a 7-day incubation, with or without (untreated control) different phytochemicals.

Group	Concentration (μg/g) ^a^
Control	4.31 ± 2.24
MT	7.01 ± 3.71
SR	15.61 ± 7.08 **
SC	12.75 ± 9.96
BBR	2.20 ± 1.81
BBR-MT	ND
BBR-SR	ND
BBR-SC	ND

^a^ Mean ± SD, *n* = 5. ND, not detected (below the method LOD of 0.5 μg/g). ** Significant difference at *p* < 0.01.

## Data Availability

The original contributions presented in this study are included in the article/Supplementary Material. Further inquiries can be directed to the corresponding authors.

## Supplementary Materials

The following supporting information can be downloaded at: https://www.mdpi.com/article/10.3390/toxins18080348/s1, Figure S1: Representative qRT-PCR amplification curves for *TRI101* (A), *TRI13* (B), *TRI1* (C), *ERG25* (D), *ERG28* (E) and *FgActin* (F). The green lines represent the untreated control group, and the blue lines represent the treated group; Table S1: Summary of key DEGs associated with ergosterol biosynthesis (*ERG*) and DON production (*TRI*); Table S2: Experimentally determined maximum soluble concentrations of the seven phytochemicals in water at room temperature; Table S3: Concentration ranges and original plant sources of fourteen phytochemicals used to evaluate the susceptibility of *Fusarium graminearum* strain PH-1 in potato dextrose broth; Table S4: Concentrations of four phytochemicals in PDA plate, PDB medium, pig feed matrix, and TBI medium used in the inhibition assays of *F. graminearum* strain PH-1; Table S5: qRT-PCR primers used in this study.

## Abbreviations

The following abbreviations are used in this manuscript:

*F. graminearum*	*Fusarium graminearum*
DON	Deoxynivalenol
QAs	Quinolizidine alkaloids
MT	Matrine
MIC	Minimum inhibitory concentration
MFC	Minimal fungicidal concentration
SR	Sophoridine
SC	Sophocarpine
BBR	Berberine hydrochloride
PDA	Potato dextrose agar
TBI	Toxigenic biosynthesis induction
HPLC	High-performance liquid chromatography
RSD	Relative standard deviation
*LOD*	Limits of detection
*LOQ*	Limits of quantification
DEG	Differentially expressed gene
PCA	Principal component analysis
qRT-PCR	Quantitative real-time polymerase chain reaction
KEGG	Kyoto Encyclopedia of Genes and Genomes
GO	Gene Ontology
BP	Kiological process
CC	Cellular component
MF	Molecular function
G6P	Glucose-6-phosphate
PDB	Potato dextrose broth
TLC	Thin layer chromatography
OMT	Oxymatrine
OSC	Ooxysophocarpine
CYT	Cytisine
SPT-S·5H_2_O	(-)-sparteine sulfate pentahydrate
GA	Gallic acid
RA	Rosmarinic acid
PF	Paeoniflorin
CGA	Chlorogenic acid
PAL	Palmatine
PSB4	Pulsatilla saponin B4
CMC	Carboxymethyl cellulose
YEPD	Yeast Extract Peptone Dextrose
FPKM	Fragments Per Kilobase Million
SD	Standard deviation

## References

[1] Lipps S., Bohn M., Rutkoski J., Butts-Wilmsmeyer C., Mideros S., Jamann T. (2025). Comparative Review of *Fusarium graminearum* Infection in Maize and Wheat: Similarities in Resistance Mechanisms and Future Directions. Mol. Plant Microbe Interact..

[2] Pestka J.J. (2010). Deoxynivalenol: Mechanisms of action, human exposure, and toxicological relevance. Arch. Toxicol..

[3] Tu Y., Liu S., Cai P., Shan T. (2023). Global distribution, toxicity to humans and animals, biodegradation, and nutritional mitigation of deoxynivalenol: A review. Compr. Rev. Food Sci. Food Saf..

[4] Zhao L., Zhang L., Xu Z., Liu X., Chen L., Dai J., Karrow N.A., Sun L. (2021). Occurrence of Aflatoxin B1, deoxynivalenol and zearalenone in feeds in China during 2018–2020. J. Anim. Sci. Biotechnol..

[5] Brooks P.H., Pluske J.R., Le Dividich J., Verstegen M.W.A. (2003). Liquid feeding as a means to promote pig health. The Weaner Pig: Nutrition and Management.

[6] Jiang J., Chen D., Yu B., He J., Yu J., Mao X., Huang Z., Luo Y., Luo J., Zheng P. (2019). Improvement of growth performance and parameters of intestinal function in liquid fed early weanling pigs. J. Anim. Sci..

[7] Cullen J.T., Lawlor P.G., Viard F., Lourenco A., Gómez-Mascaraque L.G., O’Doherty J.V., Cormican P., Gardiner G.E. (2024). Optimising the hygiene of a liquid feeding system to improve the quality of liquid feed for pigs. Sci. Rep..

[8] Pestka J.J. (2007). Deoxynivalenol: Toxicity, mechanisms and animal health risks. Anim. Feed Sci. Technol..

[9] Halawa A., Dänicke S., Kersten S., Breves G. (2013). Intestinal transport of deoxynivalenol across porcine small intestines. Arch. Anim. Nutr..

[10] Sun Y., Jiang J., Mu P., Lin R., Wen J., Deng Y. (2022). Toxicokinetics and metabolism of deoxynivalenol in animals and humans. Arch. Toxicol..

[11] Sumarah M.W. (2022). The Deoxynivalenol Challenge. J. Agric. Food Chem..

[12] Kihal A., Rodríguez-Prado M., Calsamiglia S. (2022). The efficacy of mycotoxin binders to control mycotoxins in feeds and the potential risk of interactions with nutrient: A review. J. Anim. Sci..

[13] Kihal A., Rodríguez-Prado M.E., Cristofol C., Calsamiglia S. (2021). Short Communication: Quantification of the Effect of Mycotoxin Binders on the Bioavailability of Fat-Soluble Vitamins In Vitro. Animals.

[14] Afsah-Hejri L., Hajeb P., Ehsani R.J. (2020). Application of ozone for degradation of mycotoxins in food: A review. Compr. Rev. Food Sci. Food Saf..

[15] Cui X., Zhu Q.J., Hou R., Wan J. (2022). Antibacterial Activity and Mechanism of Eugenol, Carvacrol and Thymol against *Fusarium graminearum*. Food Sci..

[16] Yang C.J., Li H.X., Wang J.R., Zhang Z.J., Wu T.L., Liu Y.Q., Tang C., Chu Q.R., Du S.S., He Y.H. (2022). Design, synthesis and biological evaluation of novel evodiamine and rutaecarpine derivatives against phytopathogenic fungi. Eur. J. Med. Chem..

[17] Li Y., Zhao M., Zhang Z. (2021). Quantitative proteomics reveals the antifungal effect of canthin-6-one isolated from Ailanthus altissima against *Fusarium oxysporum* f. sp. *cucumerinum* in vitro. PLoS ONE.

[18] Wei J., Bi Y., Xue H., Wang Y., Zong Y., Prusky D. (2020). Antifungal activity of cinnamaldehyde against *Fusarium sambucinum* involves inhibition of ergosterol biosynthesis. J. Appl. Microbiol..

[19] Cely-Veloza W., Quiroga D., Coy-Barrera E. (2022). Quinolizidine-Based Variations and Antifungal Activity of Eight *Lupinus* Species Grown under Greenhouse Conditions. Molecules.

[20] Cely-Veloza W., Yamaguchi L., Quiroga D., Kato M.J., Coy-Barrera E. (2023). Antifungal activity against Fusarium oxysporum of quinolizidines isolated from three controlled-growth Genisteae plants: Structure-activity relationship implications. Nat. Prod. Bioprospecting.

[21] Audenaert K., Callewaert E., Höfte M., De Saeger S., Haesaert G. (2010). Hydrogen peroxide induced by the fungicide prothioconazole triggers deoxynivalenol (DON) production by *Fusarium graminearum*. BMC Microbiol..

[22] Ding J., Yan Z., Peng L., Li J., Yang F., Zheng D. (2024). Inhibitory effects of berberine on fungal growth, biofilm formation, virulence, and drug resistance as an antifungal drug and adjuvant with prospects for future applications. World J. Microbiol. Biotechnol..

[23] Klinglmayr C., Nöbauer K., Razzazi-Fazeli E., Cichna-Markl M. (2010). Determination of deoxynivalenol in organic and conventional food and feed by sol-gel immunoaffinity chromatography and HPLC-UV detection. J. Chromatogr. B.

[24] Zhu Y., Hassan Y.I., Shao S., Zhou T. (2018). Employing immuno-affinity for the analysis of various microbial metabolites of the mycotoxin deoxynivalenol. J. Chromatogr. A.

[25] Liu Z., Jian Y., Chen Y., Kistler H.C., He P., Ma Z., Yin Y. (2019). A phosphorylated transcription factor regulates sterol biosynthesis in *Fusarium graminearum*. Nat. Commun..

[26] Liu X., Jiang J., Yin Y., Ma Z. (2013). Involvement of FgERG4 in ergosterol biosynthesis, vegetative differentiation and virulence in *Fusarium graminearum*. Mol. Plant Pathol..

[27] Yun Y., Yin D., Dawood D.H., Liu X., Chen Y., Ma Z. (2014). Functional characterization of FgERG3 and FgERG5 associated with ergosterol biosynthesis, vegetative differentiation and virulence of *Fusarium graminearum*. Fungal Genet. Biol..

[28] Cardoza R.E., Vizcaíno J.A., Hermosa M.R., Sousa S., González F.J., Llobell A., Monte E., Gutiérrez S. (2006). Cloning and characterization of the erg1 gene of Trichoderma harzianum: Effect of the erg1 silencing on ergosterol biosynthesis and resistance to terbinafine. Fungal Genet. Biol..

[29] Xiao H., Zhang Y., Li Q. (2025). 2-Hydroxy-4-methoxybenzaldehyde (HMB) disrupts ergosterol biosynthesis, redox metabolism, and DON biosynthesis of *Fusarium graminearum* revealed by transcriptome analysis. Front. Microbiol..

[30] Brown D.W., McCormick S.P., Alexander N.J., Proctor R.H., Desjardins A.E. (2001). A genetic and biochemical approach to study trichothecene diversity in Fusarium sporotrichioides and *Fusarium graminearum*. Fungal Genet. Biol..

[31] McCormick S.P., Harris L.J., Alexander N.J., Ouellet T., Saparno A., Allard S., Desjardins A.E. (2004). *Tri1* in *Fusarium graminearum* encodes a P450 oxygenase. Appl. Environ. Microbiol..

[32] Kasahara E., Kitamura Y., Katada M., Mizuki M., Okumura N., Sano T., Koizumi Y., Maeda K., Takahashi-Ando N., Kimura M. (2024). Attempting to Create a Pathway to 15-Deacetylcalonectrin with Limited Accumulation in Cultures of *Fusarium Tri3* Mutants: Insight into Trichothecene Biosynthesis Machinery. Int. J. Mol. Sci..

[33] Atanasova-Penichon V., Legoahec L., Bernillon S., Deborde C., Maucourt M., Verdal-Bonnin M.N., Pinson-Gadais L., Ponts N., Moing A., Richard-Forget F. (2018). Mycotoxin Biosynthesis and Central Metabolism Are Two Interlinked Pathways in *Fusarium graminearum*, as Demonstrated by the Extensive Metabolic Changes Induced by Caffeic Acid Exposure. Appl. Environ. Microbiol..

[34] Zhou Z., Zhang J., Lu F., Duan Y., Zhou M. (2021). Glucose-6-Phosphate Isomerase FgGPI, a β2 Tubulin-Interacting Protein, Is Indispensable for Fungal Development and Deoxynivalenol Biosynthesis in *Fusarium graminearum*. Phytopathology.

[35] Masi A., Mach R.L., Mach-Aigner A.R. (2021). The Pentose Phosphate Pathway in Industrially Relevant Fungi: Crucial Insights for Bioprocessing. Appl. Microbiol. Biotechnol..

[36] Li Y., Li J., Li Y., Wang X.X., Cao A.C. (2013). Antimicrobial constituents of the leaves of *Mikania micrantha* H. B. K. PLoS ONE.

[37] Liu X., Ouyang C., Wang Q., Li Y., Yan D., Yang D., Guo M., Cao A. (2016). Evaluation of antibacterial and antifungal properties of 9-oxo-10,11-dehydroageraphorone extracted from *Eupatorium adenophorum*. J. Plant Dis. Prot..

[38] Chen S., Zhou Y., Chen Y., Gu J. (2018). fastp: An ultra-fast all-in-one FASTQ preprocessor. Bioinformatics.

[39] Kim D., Langmead B., Salzberg S.L. (2015). HISAT: A fast spliced aligner with low memory requirements. Nat. Methods.

[40] Anders S., Pyl P.T., Huber W. (2015). HTSeq--a Python framework to work with high-throughput sequencing data. Bioinformatics.

[41] Love M.I., Huber W., Anders S. (2014). Moderated estimation of fold change and dispersion for RNA-seq data with DESeq2. Genome Biol..

[42] The Gene Ontology Consortium (2019). The Gene Ontology Resource: 20 years and still GOing strong. Nucleic Acids Res..

[43] Kanehisa M., Araki M., Goto S., Hattori M., Hirakawa M., Itoh M., Katayama T., Kawashima S., Okuda S., Tokimatsu T. (2008). KEGG for linking genomes to life and the environment. Nucleic Acids Res..

[44] Subramanian A., Tamayo P., Mootha V.K., Mukherjee S., Ebert B.L., Gillette M.A., Paulovich A., Pomeroy S.L., Golub T.R., Lander E.S. (2005). Gene set enrichment analysis: A knowledge-based approach for interpreting genome-wide expression profiles. Proc. Natl. Acad. Sci. USA.

