# Supplementary material for: Enhanced Inhibition of Fusarium graminearum Growth and Deoxynivalenol Production by Quinolizidine Alkaloids Combined with Berberine: In Laboratory Culture Media and Pig Feed
Source: Toxins (Basel). 2026 Aug 15;18(8):348. doi: 10.3390/toxins18080348 (PMC13517371; doi:10.3390/toxins18080348)
Supplement: Supplementary file 1 [file toxins-18-00348-s001.zip › toxins-4441098-supplementary.pdf]

Supplementary Materials: Enhanced Inhibition of *Fusarium graminearum* Growth and Deoxynivalenol Production by Quinolizidine Alkaloids Combined with Berberine: in Laboratory Culture Media and Pig Feed

Mengyang Li, Penghua Jian, Jiayi Liu, Yiwei Jia, Hui Deng, Lingxian Yi, Nan Zhuang, Zelin Hong, Daojin Yu and Bo Yang

Table S1. Summary of key DEGs associated with ergosterol biosynthesis (*ERG*) and DON production (*TRI*).

Gene ID	Symbol	Functional description	MT vs Control (log_2FC / q-value)	SR vs Control (log_2FC / q-value)	SC vs Control (log_2FC / q-value)	BBR vs Control (log_2FC / q-value)	BBR-MT vs Control (log_2FC / q-value)	BBR-SR vs Control (log_2FC / q-value)	BBR-SC vs Control (log_2FC / q-value)
FGSG_00071	*TRI1*	Cytochrome P450 monooxygenase TRI1	-	-	-	-	-1.18 / 1.3×10^{-7}	-1.30 / 1.8×10^{-10}	-
FGSG_03535	*TRI4*	Cytochrome P450 monooxygenase TRI4	-	-	-	-	3.08 / 2.3×10^{-3}	2.44 / 3.2×10^{-2}	2.52 / 1.3×10^{-2}
FGSG_03533	*TRI7*	Acetyltransferase TRI7	-	-	-	-	2.77 / 1.3×10^{-2}	3.04 / 3.9×10^{-3}	-
FGSG_03846	*TRI8*	Trichothecene C-3 esterase	-	1.17 / 7.1×10^{-13}	1.10 / 8.5×10^{-14}	-	-	-	-
FGSG_03538	*TRI10*	Trichothecene biosynthesis transcription regulator TRI10	-	-	-	-	2.66 / 6.9×10^{-4}	2.39 / 3.2×10^{-3}	-
FGSG_07765	*TRI11*	Trichothecene C-15 hydroxylase	-1.90 / 3.0×10^{-2}	-	-	-	-	-	-2.66 / 1.8×10^{-3}
FGSG_02343	*TRI12*	Trichothecene efflux pump TRI12	-	-	-	-	-	-	3.31 / 2.8×10^{-4}
FGSG_03541	*TRI12*	Trichothecene efflux pump TRI12	-	-	-	-	-1.63 / 1.8×10^{-8}	-	-
FGSG_03542	*TRI13*	Cytochrome P450 monooxygenase TRI13	-2.12 / 3.7×10^{-2}	1.93 / 3.0×10^{-4}	-	4.02 / 1.6×10^{-28}	-2.90 / 3.3×10^{-3}	-6.28 / 1.4×10^{-5}	-5.38 / 2.0×10^{-4}
FGSG_03860	*TRI13*	Cytochrome P450 monooxygenase TRI13	-	-	-	-	-1.17 / 2.8×10^{-15}	-	-
FGSG_106	*TRI13*	Cytochrome P450	-	-	-	-	7.22 / 6.7×10^{-8}	7.59 / 3.8×10^{-9}	5.34 / 9.1×10^{-4}

29		monooxygenase TRI13							
FGSG_03543	*TRI14*	Core trichothecene cluster (CTC) protein 14	-	$1.28 / 3.4\times10^{-11}$	-	$1.14 / 5.4\times10^{-7}$	$-1.33 / 3.5\times10^{-7}$	-	$-1.93 / 1.9\times10^{-13}$
FGSG_11025	*TRI15*	Core trichothecene cluster (CTC) protein 15	$3.65 / 2.1\times10^{-145}$	$2.22 / 2.5\times10^{-38}$	-	-	$2.93 / 8.0\times10^{-97}$	$1.13 / 3.3\times10^{-13}$	-
FGSG_03881	*TRI15*	Core trichothecene cluster (CTC) protein 15	-	-	-	$-1.86 / 8.9\times10^{-58}$	-	-	$-1.07 / 1.7\times10^{-33}$
FGSG_04692	*TRI101*	Trichothecene 3-O-acetyltransferase TRI101	$-1.06 / 9.1\times10^{-14}$	-	-	$1.25 / 7.3\times10^{-25}$	$-1.76 / 6.5\times10^{-37}$	$-2.27 / 4.3\times10^{-45}$	-
FGSG_07896	*TRI101*	Trichothecene 3-O-acetyltransferase TRI101	-	-	-	-	$3.53 / 3.6\times10^{-159}$	$3.59 / 2.0\times10^{-152}$	$2.21 / 2.3\times10^{-53}$
FGSG_06215	*ERG1*	Squalene epoxidase ERG1	-	-	-	-	$1.35 / 3.5\times10^{-53}$	-	-
FGSG_02502	*ERG3A*	Delta (7)-sterol 5(6)-desaturase ERG3A	-	-	-	$1.74 / 3.3\times10^{-33}$	$-1.02 / 3.2\times10^{-13}$	$-1.62 / 2.1\times10^{-23}$	$-2.26 / 1.1\times10^{-64}$
FGSG_04994	*ERG3B*	Delta (7)-sterol 5(6)-desaturase ERG3B	-	-	-	-	$2.50 / 5.3\times10^{-129}$	$1.80 / 9.8\times10^{-49}$	-
FGSG_10003	*ERG4*	Delta (24(24(1)))-sterol reductase	-	-	-	$-2.17 / 1.9\times10^{-48}$	-	-	-
FGSG_03686	*ERG5B*	C-22 sterol desaturase ERG5B	-	-	-	$1.88 / 2.8\times10^{-4}$	$1.71 / 8.0\times10^{-5}$	$1.49 / 2.03\times10^{-3}$	$1.51 / 2.4\times10^{-3}$
FGSG_05740	*ERG6B*	Sterol 24-C-methyltransferase ERG6B	$2.40 / 1.9\times10^{-2}$	-	-	$2.89 / 1.1\times10^{-3}$	$7.32 / 2.9\times10^{-46}$	$6.41 / 5.5\times10^{-34}$	$6.04 / 1.4\times10^{-26}$
FGSG_09381	*ERG9*	Squalene synthase ERG9	-	-	-	-	$2.02 / 2.3\times10^{-63}$	$1.60 / 8.9\times10^{-38}$	$1.22 / 8.0\times10^{-25}$
FGSG_09321	*ERG10*	Acetyl-CoA acetyltransferase ERG10, cytosolic	-	-	-	-	$1.84 / 2.1\times10^{-99}$	$1.14 / 3.3\times10^{-33}$	-
FGSG_05912	*ERG12*	Mevalonate kinase ERG12	-	-	-	-	$-1.06 / 3.1\times10^{-32}$	$-1.15 / 6.6\times10^{-39}$	-
FGSG_10424	*ERG19*	Diphosphomevalonate decarboxylase ERG19	-	-	-	-	$-1.11 / 3.4\times10^{-27}$	$-1.18 / 6.6\times10^{-20}$	-
FGSG_03443	*ERG24*	Delta (14)-sterol reductase ERG24	$2.72 / 8.9\times10^{-22}$	$1.89 / 1.4\times10^{-7}$	$1.15 / 9.2\times10^{-3}$	-	$2.46 / 1.6\times10^{-19}$	$1.17 / 1.5\times10^{-3}$	-
FGSG_02346	*ERG24B*	Delta (14)-sterol reductase ERG24B	$1.66 / 2.0\times10^{-47}$	$1.94 / 2.0\times10^{-66}$	$1.99 / 3.5\times10^{-64}$	3.96 / 0	5.04 / 0	4.86 / 0	$3.66 / 3.9\times10^{-304}$

FGSG_03978	*ERG25*	C-4 methylsterol oxidase ERG25	-1.31 / 1.2×10^{-13}	-	-	1.41 / 5.1×10^{-30}	-3.06 / 8.7×10^{-61}	-2.50 / 3.8×10^{-49}	-2.44 / 3.4×10^{-83}
FGSG_10666	*ERG25*	C-4 methylsterol oxidase ERG25	3.67 / 1.4×10^{-4}	2.84 / 1.4×10^{-2}	-	3.53 / 1.2×10^{-3}	4.86 / 2.5×10^{-10}	4.93 / 1.7×10^{-9}	-
FGSG_01811	*ERG26*	Sterol-4-alpha-carboxylate 3-dehydrogenase ERG26, decarboxylating	-	-	-	-	2.95 / 3.8×10^{-3}	-	-
FGSG_11714	*ERG26*	Sterol-4-alpha-carboxylate 3-dehydrogenase ERG26, decarboxylating	-	-	-	-	2.34 / 2.1×10^{-2}	1.99 / 4.8×10^{-2}	-
FGSG_05669	*ERG28*	Ergosterol biosynthetic protein 28	-	-	-	-	-1.86 / 1.5×10^{-65}	-1.92 / 2.5×10^{-54}	-1.04 / 2.6×10^{-27}

Table S2. Experimentally determined maximum soluble concentrations of the seven phytochemicals in water at room temperature.

**Phytochemical**	**Maximum soluble concentrations (mg/mL)** [a]
Gallic acid	2
Rosmarinic acid	2
Paeoniflorin	5.5
Chlorogenic acid	10
Pulsatilla saponin B4	2
Berberine hydrochloride	4
Palmatine	10

[a] These concentrations were determined as the highest concentration at which no precipitate was observed after sonication and equilibration to room temperature.

Table S3. Concentration ranges and original plant sources of fourteen phytochemicals used to evaluate the susceptibility of *Fusarium graminearum* strain PH-1 in potato dextrose broth.

**Phytochemical**	**Concentration range (mg/mL)**	**Original plant source**
Matrine	1.25–20	*Sophora flavescens* Ait., *Sophora alopecuroides* L.
Sophoridine	1–16	*Sophora flavescens* Ait., *Sophora alopecuroides* L.
Sophocarpine	1–16	*Sophora flavescens* Ait., *Sophora alopecuroides* L., *Sophora tonkinensis* Gagnep.
Oxymatrine	1–16	*Sophora flavescens* Ait., *Sophora alopecuroides* L., *Sophora tonkinensis* Gagnep.
Oxysophocarpine	1–16	*Sophora alopecuroides* L., *Sophora pachycarpa* Schrenk
Cytisine	1–16	*Sophora alopecuroides* L., *Cytisus laburnum*, *Spartium junceum* L.
(-)-sparteine sulfate pentahydrate	1–16	*Parent free base naturally occurs in Spartium junceum* L., *Cytisus scoparius*, *Lupinus* spp.
Gallic acid	0.125–2	*Rhus coriaria* L., *Terminalia chebula*
Rosmarinic acid	0.125–2	*Rosmarinus officinalis* L., *Melissa officinalis*, *Perilla frutescens*
Paeoniflorin	0.34375–5.5	*Paeonia lactiflora* Pall.
Chlorogenic acid	0.625–10	*Lonicera japonica* Thunb, *Eucommia ulmoides* Oliv.

Pulsatilla saponin B4	0.125–2	*Pulsatilla chinensis* (Bunge) Regel
Berberine hydrochloride	0.25–4	*Parent free base naturally occurs in Coptis chinensis* Franch., *Phellodendron amurense* Rupr.
Palmatine	0.625–10	*Fibraurea tinctoria* Lour., *Coptis* spp.

Table S4. Concentrations of four phytochemicals in PDA plate, PDB medium, pig feed matrix, and TBI medium used in the inhibition assays of *F. graminearum* strain PH-1.

**Group**	**Concentration (mg/mL)**
Control	-
MT	5
SR	4
SC	4
BBR	4
BBR-MT	5 (MT); 4 (BBR)
BBR-SR	4 (SR); 4 (BBR)
BBR-SC	4 (SC); 4 (BBR)

Table S5. qRT-PCR primers used in this study

Gene	Primer sequence (5'–3')	Produce size (bp)
TRI101	GCGATCGAGAAGGTGACGAT	87
	CTTCAGACGGATCATCGCGA	
TRI13	CCCAAGCACACGTGAACAAG	141
	CTTGCTGTGGACCGTTGTTG	
TRI1	TGCGCGATGGAAGGATCTAC	142
	TGTTCTCCGTAACCGAAGCC	
ERG25	ACCGGTGCCATTCATCCAAT	161
	CGCTATGTACGGCTGAGGAG	
ERG28	CTTCCTCCCTGAAGCCAAGG	133
	GCGAATAAAACGGCCGTTGT	
FgActin	TCTCTCCGACCGTATGCAGA	197
	ACTTGCGGTGAACGATTGAA	

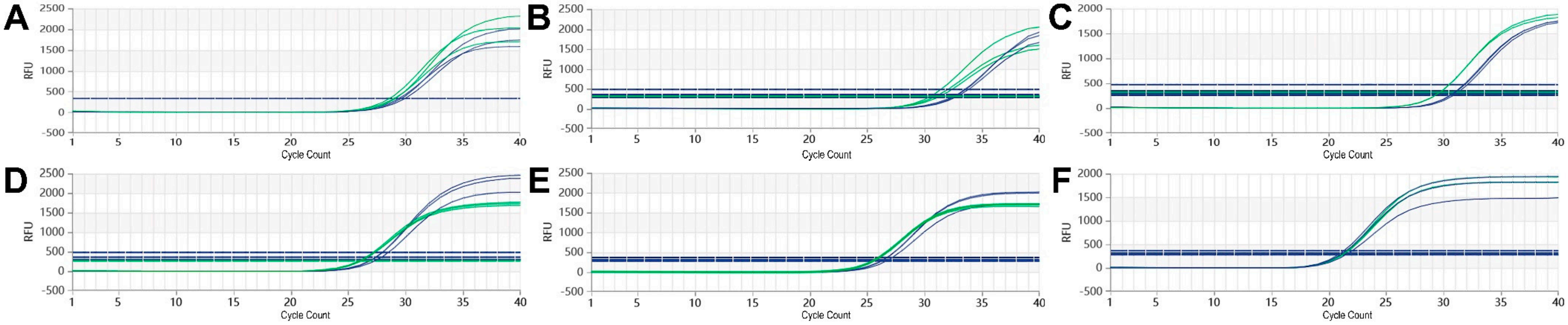

Figure S1. Representative qRT-PCR amplification curves for *TRI101* (A), *TRI13* (B), *TRI1* (C), *ERG25* (D), *ERG28* (E) and *FgActin* (F). The green lines represent the untreated control group, and the blue lines represent the treated group.
